# The CopA2-Type P_1B_-Type ATPase CcoI Serves as Central Hub for *cbb*_3_-Type Cytochrome Oxidase Biogenesis

**DOI:** 10.3389/fmicb.2021.712465

**Published:** 2021-09-13

**Authors:** Andreea Andrei, Maria Agostina Di Renzo, Yavuz Öztürk, Alexandra Meisner, Noel Daum, Fabian Frank, Juna Rauch, Fevzi Daldal, Susana L. A. Andrade, Hans-Georg Koch

**Affiliations:** ^1^Institut für Biochemie und Molekularbiologie, ZBMZ, Faculty of Medicine, Albert-Ludwigs-Universität Freiburg, Freiburg, Germany; ^2^Faculty of Biology, Albert-Ludwigs-Universität Freiburg, Freiburg, Germany; ^3^Institute for Biochemistry, Faculty of Chemistry and Pharmacy, Albert-Ludwigs-University Freiburg, Freiburg, Germany; ^4^Spemann Graduate School of Biology and Medicine (SGBM), University of Freiburg, Freiburg, Germany; ^5^Faculty of Chemistry and Pharmacy, Albert-Ludwigs-Universität Freiburg, Freiburg, Germany; ^6^Department of Biology, University of Pennsylvania, Philadelphia, PA, United States

**Keywords:** P_1B_-type ATPases, CcoI, cbb3-type cytochrome c oxidase biogenesis, SenC, *Rhodobacter capsulatus*, solid-supported membrane electrophysiology, CopZ

## Abstract

Copper (Cu)-transporting P_1B_-type ATPases are ubiquitous metal transporters and crucial for maintaining Cu homeostasis in all domains of life. In bacteria, the P_1B_-type ATPase CopA is required for Cu-detoxification and exports excess Cu(I) in an ATP-dependent reaction from the cytosol into the periplasm. CopA is a member of the CopA1-type ATPase family and has been biochemically and structurally characterized in detail. In contrast, less is known about members of the CopA2-type ATPase family, which are predicted to transport Cu(I) into the periplasm for cuproprotein maturation. One example is CcoI, which is required for the maturation of *cbb*_3_-type cytochrome oxidase (*cbb*_3_-Cox) in different species. Here, we reconstituted purified CcoI of *Rhodobacter capsulatus* into liposomes and determined Cu transport using solid-supported membrane electrophysiology. The data demonstrate ATP-dependent Cu(I) translocation by CcoI, while no transport is observed in the presence of a non-hydrolysable ATP analog. CcoI contains two cytosolically exposed N-terminal metal binding sites (N-MBSs), which are both important, but not essential for Cu delivery to *cbb*_3_-Cox. CcoI and *cbb*_3_-Cox activity assays in the presence of different Cu concentrations suggest that the glutaredoxin-like N-MBS1 is primarily involved in regulating the ATPase activity of CcoI, while the CopZ-like N-MBS2 is involved in Cu(I) acquisition. The interaction of CcoI with periplasmic Cu chaperones was analyzed by genetically fusing CcoI to the chaperone SenC. The CcoI-SenC fusion protein was fully functional *in vivo* and sufficient to provide Cu for *cbb*_3_-Cox maturation. In summary, our data demonstrate that CcoI provides the link between the cytosolic and periplasmic Cu chaperone networks during *cbb*_3_-Cox assembly.

## Introduction

Copper is an essential, yet potentially toxic micronutrient, which is required for the catalytic activity of many important enzymes, like cytochrome oxidases (Cox) or Cu-Zn superoxide dismutases. The redox properties of Cu are especially crucial for the oxygen-linked activities of these enzymes, but they also favor cytotoxic effects that can lead to the formation of hydroxyl radicals (Argüello et al., 2013; Andrei et al., 2020). Cu homeostasis is therefore tightly controlled by the coordinated action of Cu transporters and Cu chaperones to maintain a sufficient Cu quota for cuproprotein biogenesis while preventing accumulation of excess Cu in the cytosol (Rosenzweig and O'Halloran, 2000; Rensing and Grass, 2003; Banci et al., 2010a; Arguello et al., 2016). Central to this coordination are P_1B_-type ATPases, a ubiquitous class of transition metal exporters, located in the cytoplasmic membrane of bacteria, the basolateral membrane of enterocytes or the trans-Golgi network of hepatocytes and brain cells (Crisponi et al., 2010; Smith et al., 2014; Andrei et al., 2020).

Despite sequence variations, the overall structure of Cu-transporting P_1B_-type ATPases is largely conserved between the family members from different species. They usually consist of eight transmembrane helices (TM) and three cytoplasmic domains (Arguello et al., 2007). The transmembrane module contains two invariable metal binding sites (MBSs), TM-MBS1 and TM-MBS2, characterized by the presence of conserved Cys-Pro-Cys and Asp-Met-Ser motifs, respectively (Wu et al., 2008). Inactivation of any of these sites leads to the inhibition of Cu transport, and reduced ATPase activity (Gonzalez-Guerrero et al., 2008, 2009). In the cytoplasmic module, the nucleotide-binding domain (N-domain) binds ATP for the transient phosphorylation of an invariant Asp residue located in the phosphorylation domain (P-domain). The third cytoplasmic domain, the actuator domain (A-domain), is suggested to relay conformational changes from the N- and P-domains to the transmembrane module and to control the dephosphorylation of the P-domain. The ATP-dependent Cu translocation by P_1B_-ATPases largely follows the Post-Albers cycle, first described for Na^+^, K^+^-ATPases (Post et al., 1972; Palmgren and Nissen, 2011). However, due to the potential toxicity of Cu and the virtual absence of free Cu in the bacterial cytosol, the catalytic activity of P_1B_-type ATPases is thought to be further influenced by Cu transfer reactions from cytosolic Cu chaperones to their MBSs (Arguello et al., 2016).

Typically, bacteria contain more than one Cu-exporting P_1B_-type ATPase. While CopA1-type ATPases export excess Cu and primarily mediate Cu detoxification, CopA2-type ATPases provide Cu for cuproprotein biogenesis (Preisig et al., 1996; Koch et al., 2000; Gonzalez-Guerrero et al., 2010; Smith et al., 2014). This functional diversity is also reflected by distinct catalytic activities: CopA1-type ATPases have a high turnover rate and low affinity for Cu(I), while the opposite applies to CopA2-type ATPases, in line with the general concept that affinity gradients are important determinants for the cellular Cu metabolism (Banci et al., 2010c).

**Figure 1:**
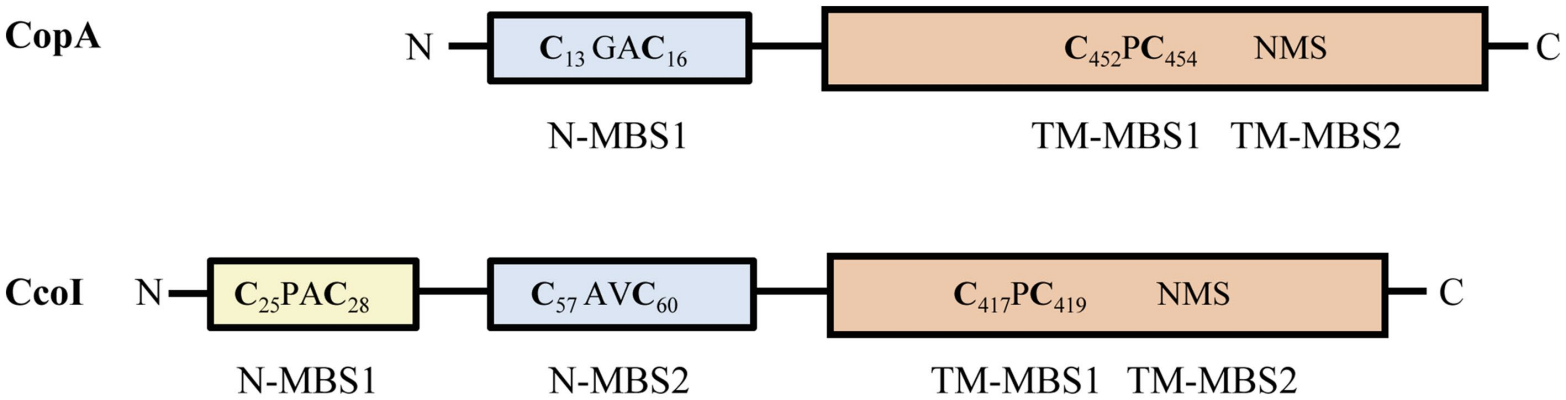
Comparison of *Rhodobacter capsulatus* CopA and CcoI. The CopZ-like CxxC Cu binding site (N-MBS1 in CopA and N-MBS2 in CcoI) is depicted in pale blue. CcoI contains an additional glutaredoxin-like MBS (CPAC, N-MBS1) at the very N-terminus, depicted in yellow. The core cytoplasmic and transmembrane domains of CcoI and CopA are depicted in orange, with the two transmembrane Cu binding sites indicated (TM-MBS1 & 2). Numbers in subscript refer to the amino acid positions in the respective protein.

In addition to their different catalytic properties, CopA1- and CopA2-type ATPases also show variations in their soluble MBSs (Figure 1), which can be located at the N-terminus (N-MBSs) or at the C-terminus (C-MBSs; Mandal and Arguello, 2003; Drees et al., 2015). In most CopA1-type ATPases, the N-MBSs are not essential for Cu-transport, but rather function as Cu-sensing domains to regulate Cu translocation rates, potentially *via* interaction with the ATP-binding N-domain and A-domain (Wu et al., 2008). The most common Cu-binding motif in N-MBSs is a Met-x-Cys-x-x-Cys motif that adopts a ferredoxin-like *βαββαβ* fold, also found in cytosolic CopZ-like Cu chaperones (Banci et al., 2003; Rodriguez-Granillo and Wittung-Stafshede, 2008; Agarwal et al., 2010). Matching Cu-binding motifs in CopA1-ATPases and their cognate chaperones appears to be important for their activity, as illustrated by the less common cupredoxin-like Cys-x-Cys-x-Met-x-Met motif of *Streptococcus pneumoniae* CopA, which is also present in its cognate chaperone CupA (Fu et al., 2013).

Little is known about the function of the N-MBSs in CopA2-type ATPases, which like CcoI, are relevant for the export of Cu required for the *cbb*_3_-type Cox in bacteria. This type of Cox is widely distributed in bacteria, but absent in eukaryotes (Ekici et al., 2012). The *cbb*_3_-type Cox of the facultative phototrophic bacterium *Rhodobacter capsulatus* has developed into a well-studied model system for dissecting bacterial Cu homeostasis, because *cbb*_3_-Cox is the only Cox present in this organism and contains only one Cu ion in its catalytic heme *b*-Cu_B_ center of subunit CcoN (Gray et al., 1994; Steimle et al., 2021). This is different from the universally conserved *aa*_3_-type Cox, which contains a second Cu center in the electron-accepting subunit II (Thompson et al., 2012). In *cbb*_3_-Cox, this subunit is replaced by the two membrane-bound *c*-type cytochromes, CcoO and CcoP (Ekici et al., 2012). The absence of CcoI in *R. capsulatus* cells prevents the formation of an active *cbb*_3_-Cox, but has no major effect on Cu sensitivity, in agreement with its dedicated function in cuproprotein biogenesis (Utz et al., 2019). CcoI is part of a complex Cu supply chain identified in *R. capsulatus* (Koch et al., 2000), which starts with Cu(II)-uptake by the major facilitator super family member CcoA (Ekici et al., 2014). Once in the cytosol, Cu(II) is reduced to Cu(I) by the membrane-bound ferredoxin-like protein CcoG. CcoG represents the first bacterial cupric reductase that reduces Cu(II) in the cytosol, and this activity is important for efficient *cbb*_3_-type Cox assembly (Marckmann et al., 2019). Cu(I) is then likely bound to the Cu chaperone CopZ. *R. capsulatus* CopZ was found to interact with both CopA and CcoI and this interaction is likely guided by the Cu concentration in the cytosol. At high Cu concentrations, CopZ primarily transfers Cu to CopA for extrusion, while at low Cu concentrations, CopZ mediates Cu transfer to CcoI for *cbb*_3_-Cox assembly (Utz et al., 2019). Interestingly, the absence of CopZ markedly increases Cu sensitivity in *R. capsulatus*, but only slightly reduces *cbb*_3_-Cox activity, indicating that Cu detoxification is the primary role of CopZ. In addition, at low Cu availability, two periplasmic Cu chaperones are involved in conveying Cu to *cbb*_3_-Cox. The membrane-bound SenC is homologous to the ScoI-family of Cu chaperones, also involved in the assembly of the Cu_A_-center in subunit II of *aa*_3_-Cox (Canonica et al., 2019a,b). SenC cooperates with the soluble, periplasmic Cu chaperone PccA and Cu transfer between both proteins has been confirmed experimentally (Trasnea et al., 2018). *In vitro* studies showed that Cu transfer between SenC and PccA works in both directions (Trasnea et al., 2018), leaving unknown whether SenC, PccA or a so far unknown protein is the primary acceptor of translocated Cu(I) in the periplasm. In the absence of PccA, *cbb*_3_-type Cox activity is reduced to approx. 30%, but when *senC* is deleted basically no *cbb*_3_-type Cox activity is present under low Cu availability (Lohmeyer et al., 2012; Trasnea et al., 2016), indicating that PccA is not essential for *cbb_3_*-Cox assembly, even at Cu-limiting conditions.

In *R. capsulatus*, CcoI is considered to be a central player in the Cu supply pathway to *cbb*_3_-Cox, yet experimental proof that it indeed transports Cu is lacking. In addition, the unusual presence of two distinct N-MBSs in CcoI raises questions about their role(s) in CcoI-mediated Cu delivery to *cbb*_3_-Cox. Finally, how CcoI cooperates with periplasmic Cu chaperones for *cbb*_3_-Cox assembly is largely unknown. In the current study, we used solid-supported membrane electrophysiology (SSM) to demonstrate the ATP-dependent Cu(I) translocation by CcoI. This method allows to monitor the ability of transporter proteins to translocate charged ions across the membrane. Furthermore, we analyzed the role of the two N-MBSs in CcoI and provide evidence that a CcoI-SenC complex is required for *cbb*_3_-Cox assembly.

## Materials and Methods

### Bacterial Strains and Growth Conditions

*Escherichia coli* C43(DE3) cells were grown aerobically in lysogeny broth (LB) at 37°C. When indicated, the medium was supplemented with 50μg/ml ampicillin or 12.5μg/ml tetracycline. *R. capsulatus* wild type and mutant strains were grown semi-aerobically on enriched yeast-peptone medium (MPYE) or Sistrom’s minimal medium (Sistrom, 1960) at 35°C, with 110rpm shaking and supplemented with appropriate antibiotics (Supplementary Table S1). For protein production, solid and liquid media were supplemented with 0.2% or 0.5% *L*-arabinose (*L*-ara), respectively. All strains and plasmids are listed in Supplementary Tables S1, S2.

### Molecular Genetic Techniques

For mutating the CcoI MBSs, the plasmid pBAD-*ccoI*-Myc/HisA, which contains *ccoI* under the control of the P_ara_ promoter (Utz et al., 2019), was used as template for PCR-mutagenesis. All primers are listed in Supplementary Table S3. This generated the plasmids pBAD-*ccoI(C_25_A/C_28_A)*-Myc/His, pBAD*-ccoI(C_57_A/C_60_A)*-Myc/His and pBAD*-ccoI(C_417_A/C_419_A)*-Myc/His, which were confirmed by sequencing. The constructs were transformed into *E. coli* C43(DE3) for protein purification. For expression in *R. capsulatus* strains, the constructs were inserted into the broad host range plasmid pRK415. First, the pBAD-*ccoI*-Myc/HisA derivatives were linearized using the pBAD-specific 4a and 4b primers (Supplementary Table S2) with 20bp 5' overlapping regions containing the BamHI restriction site and then integrated into BamHI digested pRK415 by the HiFi Gibson assembly method, as described by the manufacturer (NEB Lab, MA). The constructs were first transformed into chemically competent *E. coli* DH5-α cells, and thereafter to HB101 cells for heterologous conjugation. The pRK-*ccoI(C_25_A/C_28_A)*, pRK-*ccoI(C_57_A/C_60_A)*, and pRK-*ccoI(C_417_A/C_419_A)* constructs were confirmed by sequencing and then conjugated into *R. capsulatus* strains by triparental conjugation. CcoI production was monitored after induction with 0.5% *L*-ara and visualized by immune blotting using antibodies against the Myc and His tags.

For the chromosomal inactivation of *copZ* and *copA* genes in the *R. capsulatus* Δ*ccoI* strain, the pRK415-Δ*copZ* plasmid carrying the Δ(*copZ::Gm*) allele was introduced into *R. capsulatus* Gene Transfer Agent (GTA) overproducer strain Y262, as described before (Koch et al., 1998; Utz et al., 2019). The GTA particles carrying the Δ(*copZ::Gm*) allele were mixed with the Δ*ccoI* strain for GTA mediated interposon mutagenesis (Yen et al., 1979; Daldal et al., 1986), yielding the Δ*ccoI*Δ*copZ* double mutant. The Δ*ccoI*Δ*copA* double mutant was generated as described (Öztürk et al., Submitted). The pRK-*copA*::Kan plasmid carrying the Δ(*copA::kan*) allele (RCAP_rcc01180) in *R. capsulatus* Y262 strain (Ekici et al., 2014) provided the GTA particles used with the Δ*ccoI* strain to generate the Δ*ccoI*Δ*copA* double mutant (Supplementary Table S1).

The *ccoI*-*senC* fusion and its inactive variants *ccoI(C_417_A/C_419_A)-senC* and *ccoI-senC(C_83_A/C_87_A)* were cloned into the *L*-ara inducible expression vector pBAD-Myc/HisA. The plasmid pBAD was linearized by using the primer 5a and 5b (Supplementary Table S3) and the PCR product thus obtained was digested with DpnI to remove the template plasmid. The *ccoI* coding sequence without its stop codon was genetically fused to the *senC* coding sequence starting at Gly_2_ as its first amino acid, *via* an overlapping sequence encoding GGSG-*FLAG*-GGSG as a linker, and amplified by using the pair of primers 6a and 6b, 7a and 7b (Supplementary Table S3). Genomic DNA of *R. capsulatus* MT1131 was used as a template. For the mutated versions of *ccoI* and *senC*, the pair of primers 8a and 8b, 9a and 9b (Supplementary Table S3) were used, respectively. HiFi Gibson assembly method (NEB) was used as described above to yield pBAD-*ccoI-senC*, pBAD-*ccoI(C_417_A/C_419_A)-senC*, and pBAD*-ccoI-senC(C_83_A/C_87_A)* plasmids, which were confirmed by sequencing. These plasmids were then integrated into the broad host range plasmid pRK415, generating pRK-*ccoI-senC*, pRK-*ccoI(M)-senC*, and pRK-*ccoI-senC(M)* (*M* designating the respective mutants), which were confirmed by sequencing and transformed into chemically competent *E. coli* HB101 cells for subsequent triparental conjugation into *R. capsulatus* strains.

### CcoI Purification

*E. coli* C43(DE3) cells producing wild-type and appropriate variants of CcoI for downstream purification purposes were grown to an optical density (OD_600_) of 0.6, cooled down for 20min at 4°C and supplemented with 0.2% *L*-ara. Cultures were incubated at 30° C, 180rpm for 90min., and all downstream steps were performed on ice. Cells were harvested at 7,460 *x g* for 12min in a JLA 9.100 rotor. The pellet was washed in 50ml 50mM TeaAc buffer and recentrifuged. 10ml of resuspension buffer (50mM TeaAc, pH 7.5, 1mM EDTA, pH 8, 0.4mM aminohexanoic acid) per 10g of cells was used to resuspend the pellet. Prior to cell disruption, 2mM Pefabloc, 2mM DTT and 5 Complete Protease Inhibitor tablets (Roche, Germany) were added per 100ml of cell suspension, which was passed three times through the Emulsiflex-c3 (Avestin, Ontario, Canada) with an applied pressure of 10,000psi. After cell disruption, cell debris were removed by centrifugation for 20min in a F21 rotor at 27,000 *x g*. The supernatant was then further centrifuged at 192,800 *x g* in a Ti 50.2 rotor for 2h, to isolate the membrane fraction. Subsequently, the membranes were resuspended with a loose Dounce homogenizer pestle in 10ml dilution buffer (25mM Tris, pH 7.5, 500mM NaCl, 10mM MgCl_2_, 10% Glycerol, 2mM Pefabloc, 2mM DTT, 1 tablet of Complete Protease Inhibitor/50ml buffer) per each gram of membrane fraction. Membrane proteins were solubilized using 1% (*w/v*) DDM (ThermoFisher Scientific, Germany) in the presence of 2mM Pefabloc, 2mM DTT, and 1 tablet of complete protease Inhibitor/50ml suspension and incubated, under gentle rotation for 1h at 4°C. Solubilized proteins were separated from the insoluble material by centrifugation at 192,800 *x g*, for 45min, 4° C, using a 50.2 Ti rotor. Finally, the supernatant was spiked with imidazole pH 7.5 to reach a final concentration of 25mM and filtered through a 0.4μm sterile filter immediately before injecting it onto a 5ml HisTrap HP (High Purity) affinity column (VWR International), previously charged with Ni and equilibrated in Buffer A (dilution buffer enriched with 0.03% (*w/v*) DDM and 25mM imidazole pH 8.0). After three washing steps with increasing imidazole concentrations (25mM, 50mM, and 100mM imidazole, respectively) and removing impurities, CcoI was selectively eluted with Buffer A enriched with 0.03% (*w/v*) DDM and 300mM imidazole. The pooled CcoI fractions were then concentrated using an Amicon filter with 50kDa cut-off size (Milipore, Germany) and injected onto a size exclusion Superdex 200 16/600 column (GE Healthcare, Germany) pre-equilibrated in Buffer B (25mM Tris pH 7.5, 10mM MgCl_2_, 300mM NaCl, 5% Glycerol, 2mM DTT, 0.03% DDM). The monomeric protein peak was pooled, concentrated to 10mg/ml, as determined using the BCA assay kit (ThermoFisher Scientific, Germany) and directly used for proteoliposome reconstitutions or ATPase assays. All steps described were performed at 4°C.

### Liposome Preparation and Proteoliposome Reconstitution

Three lipid mixtures were used to prepare liposomes for CcoI reconstitution: (a) 1ml *E. coli* polar lipids (Polar), (b) 15mg/ml *E. coli* polar lipids supplemented with 4mg/ml phosphatidyl-choline (Polar + PC) and (c) a 1ml mixture of pure lipids (3.5mg/ml DSPC (18:0 1,2-distearoyl-sn-glycero-3-phosphatidyl-choline), 3.5mg/ml PE (16,1 1,2-dipalmitoleoyl-sn-glycero-3-phosphatidyl-ethanolamine), 2mg/ml PG (18:0–18:1 1-stearoyl-2-oleoyl-sn-glycero-3-phospho-(1'-rac-glycerol), 1mg/ml DGTS (1,2-dipalmitoyl-sn-glycero-3-O-4'-(N,N,N-trimethyl)-homoserine); all obtained from Avanti Polar Lipids, Alabaster, United States). The pure lipid mixture was designed to better mimic the native *R. capsulatus* membrane lipid composition (Imhoff, 1991; Aygun-Sunar et al., 2006). The lipids were dissolved in chloroform and mixed in a round flask. The chloroform was then evaporated using a rotary evaporator operating under a 100–150mbar vacuum for 1h, at room temperature. The resulting dried lipid film was hydrated using 2.5ml liposome buffer (25mM Tris-HCl pH 7.5, 300mM KCl) to a final lipid concentration of 10mg/ml and resuspended for 90min in the rotary evaporator by rotation, without vacuum. To obtain large unilamellar vesicles, the lipid suspensions were extruded 21 times through a 0.4μm polycarbonate membrane (Whatman^®^; Merck, Germany) using a mini-extruder (Avanti Polar Lipids, Alabaster, Unied States). 250μl aliquots of the different liposomes were flash frozen in liquid nitrogen and stored at −80°C until further use.

For CcoI reconstitution, 250μl of a chosen liposome suspension were slowly thawed and destabilized by the addition of 50μl of 10% (*v/v*) Triton X-100, for 1h at room temperature, prior to protein incorporation. To prepare proteoliposomes with a lipid-to-protein ratio (LPR) of 5:1, 60μl of pure CcoI (10mg/ml) were added to detergent-destabilized liposomes. The suspension was incubated for 30min at room temperature and the detergent was removed using bio-beads (Bio-Rad Laboratories, Germany) and proteoliposome buffer (25mM Tris–HCl pH 7.5, 300mM KCl, 5mM MgCl_2_), following the supplier instructions. The 2× 1h incubation with activated bio-beads were performed at room temperature on a rocking platform, while the third incubation step was carried out at 4°C, overnight. The final proteoliposome suspensions were aliquoted and, if not immediately used, flash frozen in liquid N_2_ and stored at −80°C.

### Solid Supported Membrane Electrophysiology

To determine the ability of CcoI to transport Cu(I), solid-supported membrane (SSM) electrophysiology was employed (Schulz et al., 2008; Grewer et al., 2013; Wacker et al., 2014; Rycovska-Blume et al., 2015; Bazzone et al., 2017; Tadini-Buoninsegni, 2020). All measurements were performed on a SURFE^2^R-N1 instrument (Nanion Technologies, Munich, Germany). For these experiments, 3mM diameter gold electrodes were coated with 50μl of 0.5mM 1-octadecanethiol in isopropanol, for 45min at room temperature. Then, 1.5μl of a lipid suspension (16.5mg/ml diphytanoyl phosphatidylcholine and 0.28mg/ml octadecylamine, in *n*-decane; Avanti Lipids) was added and covered in 50μl of proteoliposome buffer (25mM Tris-HCl pH 7.5, 300mM KCl, 5mM MgCl_2_). Finally, to establish the sensor unit, 10μl of liposomes (negative control) or proteoliposomes were added to adsorb onto the hybrid bilayer-coated electrodes by 1h incubation at room temperature, followed by a 30min centrifugation step at 2,500 *x g*, 20 °C. Once inside the instrument, the sensor is subject to a rapid flux (100μl/s) and exchange of solutions (Bazzone et al., 2013, 2017; Wacker et al., 2014). Typically, exchange cycles between a non-activating (NA), activating (A), and again NA-solutions, lasting 2s each, are applied. The two solutions are ionically equivalent, but the molecular species thought to trigger the electrogenic event is only present in the A-solution while the NA-solution should minimize solution exchange and lipid interaction artifacts, and also brings the system back to baseline (Toyoshima, 2009; Mattle et al., 2015; Smith et al., 2015; Bazzone et al., 2017; Gantner, 2017). In our case, on top of containing a stabilizing buffer for the protein and enough salts to reduce artifacts (Bazzone et al., 2013, 2017), the NA- and A-solutions also contained MgCl_2_ (to stabilize ATP), cysteine (to maintain the Cu(I) oxidation state and deliver it to the ATPase; Yang et al., 2007) and ascorbic acid to reduce Cu(II) to Cu(I).

In SSM-electrophysiology, the establishment of a compound membrane between the adsorbed vesicles and the hybrid bilayer on the gold electrode surface (the measuring electrode) allows recording transient currents *via* capacitive coupling (Bazzone et al., 2017). In brief, the rapid exchange of solutions establishes a membrane potential across the vesicle membrane that acts as driving force for protein-mediated electrogenic events. Typically, the analysis of such transient currents is done upon perfusion of the system from a NA- to A-solution. The peak of the transient reflects the number of charges that participate in the electrogenic event and its decay in a function of the electrogenic event itself and the amount of protein that is present in the system (Bazzone et al., 2017; Gantner, 2017). The transient currents were initially analyzed using the SURFE2R-N1 instrument specific analysis software (Nanion) and further processed in OriginPro 2020 and Excel.

### ATPase Assay

The ATPase activity of CcoI was determined by using ^33^P-ATP (Hartmann Analytic, Braunschweig, Germany) as substrate (Wenk et al., 2012). Purified CcoI or its variants were incubated with 10–100μm ^33^P-ATP solution in a final volume of 20μl reaction buffer (50mM Tris pH 7.5, 300mM NaCl, 10mM MgCl2, 0.03% DDM, 10% glycerol). The amount of protein to be used was determined by titration of 1–16μM final protein concentration in the reaction mixture. To measure the effect of Cu(I) on the ATPase activity of CcoI, Cu(II) was reduced to Cu(I) with 10mM ascorbic acid and titrated to the reaction mixture. The reaction was incubated for 15min at 35° C, with gentle shaking. The released inorganic phosphate was separated from the reaction mixture by mixing with 800μl charcoal solution (20mM phosphoric acid, 5% charcoal). After 10min incubation on ice, the charcoal was pelleted in a precooled table-top centrifuge for 12min at 17,130 *x g* maximum speed. To determine the amount of hydrolyzed ^33^PO_4_, 200μl of the supernatant was mixed with 3ml scintillation liquid and measured with a scintillation counter (PerkinElmer Life Sciences, Waltham, United states). As controls, blank measurements were performed without protein and subtracted from the experimental values.

### Preparation of ICM and Blue Native PAGE Analyses

For preparation of inverted cytoplasmic membranes (ICMs) of *R. capsulatus* strains, previously published protocols were used (Koch et al., 1998; Pawlik et al., 2010). Native complexes were investigated on Blue Native (BN) PAGE as described in (Utz et al., 2019) and antibodies against c-Myc (clone 9E10, Sigma Aldrich) and polyclonal antibodies against CcoN (Koch et al., 1998) were used.

### *cbb_3_*-Cox Activity Assays (DMPD and TMPD)

For dimethyl-*p*-phenylendiamine (DMPD) oxidation (Marckmann et al., 2019), 10^8^ cells with an OD_685_ 0.8–1 were collected from each strain and mixed with 1mM DMPD in Buffer D (25mM Tris pH 7.5, 150mM NaCl) to a final volume of 1ml. DMPD oxidation was monitored photometrically at 554nm for at least 4min. The DMPD oxidation in wild type MT1131 was set to 100% *cbb_3_*-Cox activity. The TMPD (*N, N, N, N*-tetramethyl-*p*-phenylendiamine) oxidation assay for determining *cbb_3_*-Cox activity was performed using ICMs. 10μg of total protein in ICMs were mixed with 1mM TMPD and adjusted to a final volume of 1ml with Buffer D. TMPD oxidation was monitored photometrically for at least 30s at 611nm. Cox-independent TMPD oxidation was monitored in the presence of 200μM sodium-cyanide, a specific inhibitor of *cbb*_3_-Cox, and subtracted from the cyanide-free sample. Data processing was performed as described for the DMPD assay.

### Reproducibility Statement

All experiments were performed multiple times with independent biological and technical replicates as indicated in the legends to the figures. The mean values and standard deviations (SD) were calculated using the *Excel* and *Graphpad prism* softwares. For statistical analyses, a Student unpaired two-way t-test with the Satterthwaite correction was performed (Welch-test).^1^ Probability values (values of *p*) are indicated in the legends to the Figures. A value of *p* >0.05 was generally considered to be not significant (n.s.). To obtain statistically meaningful SSM data, a minimum of five sensors were analyzed per proteoliposome batch and at least two proteoliposome preparations for a given protein/variant were used. Reported peak current values represent the average of a minimum of three measurements per sensor and the error bars represent the standard deviation of the mean. At the end of a given set of experiments, the stability of the protein used was confirmed by comparing the final electrogenic signal obtained to that recorded under the initial conditions. Only signal losses less than 20% were accepted.

## Results

### The P_1B_-Type ATPase CcoI Drives Electrogenic Cu(I) Efflux For *cbb*_3_-Cox Maturation

CcoI shares high sequence similarity with P_1B_-type ATPases, which constitute a conserved family of ATP-dependent Cu(I) export proteins (Arguello et al., 2007; Smith et al., 2014). CcoI contains four putative MBSs (Figure 1; Supplementary Figure S1). The cytosolically exposed N-terminus contains two MBSs: the glutaredoxin-like CPAC motif (N-MBS1; Hatori and Lutsenko, 2016) is present in CcoI-like P_1B_-type ATPases, but absent in CopA-like P_1B_-type ATPases (Figure 1), whereas the CAVC motif (N-MBS2) is conserved in both CopA- and CcoI-like ATPases and is homologous to the Cu-binding motif of the CopZ-like copper chaperones (Utz et al., 2019; Andrei et al., 2020). Two other MBSs are present in the membrane-integral domain of CcoI (Figure 1). The CPC motif (TM-MBS1) is located in the transmembrane helix (TM) 4 and the NMS motif (TM-MBS2) is formed by an asparagine residue from TM5 together with methionine and serine residues from TM8. Both TM-MBSs are conserved among Cu-transporting P_1B_-ATPases (Arguello et al., 2007; Smith et al., 2014) and are necessary to drive the outward transport of copper (Mandal et al., 2004).

Cu transport by CopA-like P_1B_-type ATPases has been extensively studied using different methods (Gonzalez-Guerrero et al., 2010; Völlmecke et al., 2012; Drees et al., 2015; Abeyrathna et al., 2020; Tadini-Buoninsegni, 2020), but similar information on CcoI-type ATPases is limited. Cu transport by CopA1 and CopA2 of *Pseudomonas aeruginosa* has been determined by using *E. coli* expressed proteins and monitoring ^64^Cu accumulation in membrane vesicles (Gonzalez-Guerrero et al., 2010). This allowed for identifying important kinetic differences between the two types of Cu transporting ATPases (Raimunda et al., 2011). Yet, the activity of a CopA2-type ATPase has not been studied so far using a reconstituted system devoid of any other transporter, which might interfere with data interpretation.

Earlier work demonstrated that the absence of CcoI results in an inactive and unstable *cbb*_3_-Cox (Koch et al., 2000; Utz et al., 2019). The *cbb*_3_-Cox deficiency in the Δ*ccoI* strain could not be complemented by increasing the external Cu concentration in the medium, or by expressing a plasmid-borne copy of *copA*, encoding for the second Cu-exporting P_1B_-type ATPase present in the *R. capsulatus* membrane (Ekici et al., 2014; Utz et al., 2019). In order to validate the hypothesis that CcoI is involved in the transport of Cu for *cbb*_3_-Cox maturation, we employed solid-supported membrane electrophysiology. This technique allows monitoring protein-mediated electrogenic events in a controlled *in vitro* setup (Wacker et al., 2014; Bazzone et al., 2017). Thus, *R. capsulatus* CcoI containing a C-terminal Myc-His tag was expressed in *E. coli* C43(DE3) cells, purified to homogeneity (Supplementary Figure S2), and reconstituted into liposomes (CcoI-LPs) at a 5:1 lipid-toprotein ratio (LPR). After adsorption of the CcoI-LPs to the sensor surface, the electrogenic response of the protein was monitored as transient currents established over time. The use of purified CcoI, reconstituted into liposomes, offers the unique advantage of monitoring its Cu translocation properties in the absence of any other protein.

To optimize the background signal-to-noise ratio of the SSM measurements and minimize any unspecific interaction of Cu(I) with the polar head group of the lipids, various lipid and buffer components were tested (Supplementary Figure S3). Three lipid mixtures were used to generate liposomes (LPs): (a) *E. coli* polar lipids (Avanti Polar Lipids) (b) *E. coli* polar lipids supplemented with the neutral phospholipid PC (phosphatidylcholine) in a 3:2 ratio, and (c) a mixture of pure phospholipids, containing 18:0 PC (DSPC; 1,2-distearoyl-sn-glycero-3-phosphocholine), 16:1 PE (1,2-dipalmitoleoyl-sn-glycero-3-phosphoethanolamine), 18:0–18:1 PG (1-stearoyl-2-oleoyl-sn-glycero-3-phospho-(1'-rac-glycerol; sodium salt)), DGTS (1,2-dipalmitoyl-sn-glycero-3-O-4'-(N,N,N-trimethyl)-homoserine) in a 2:3.5:3.5:1 ratio.

**Figure 2:**
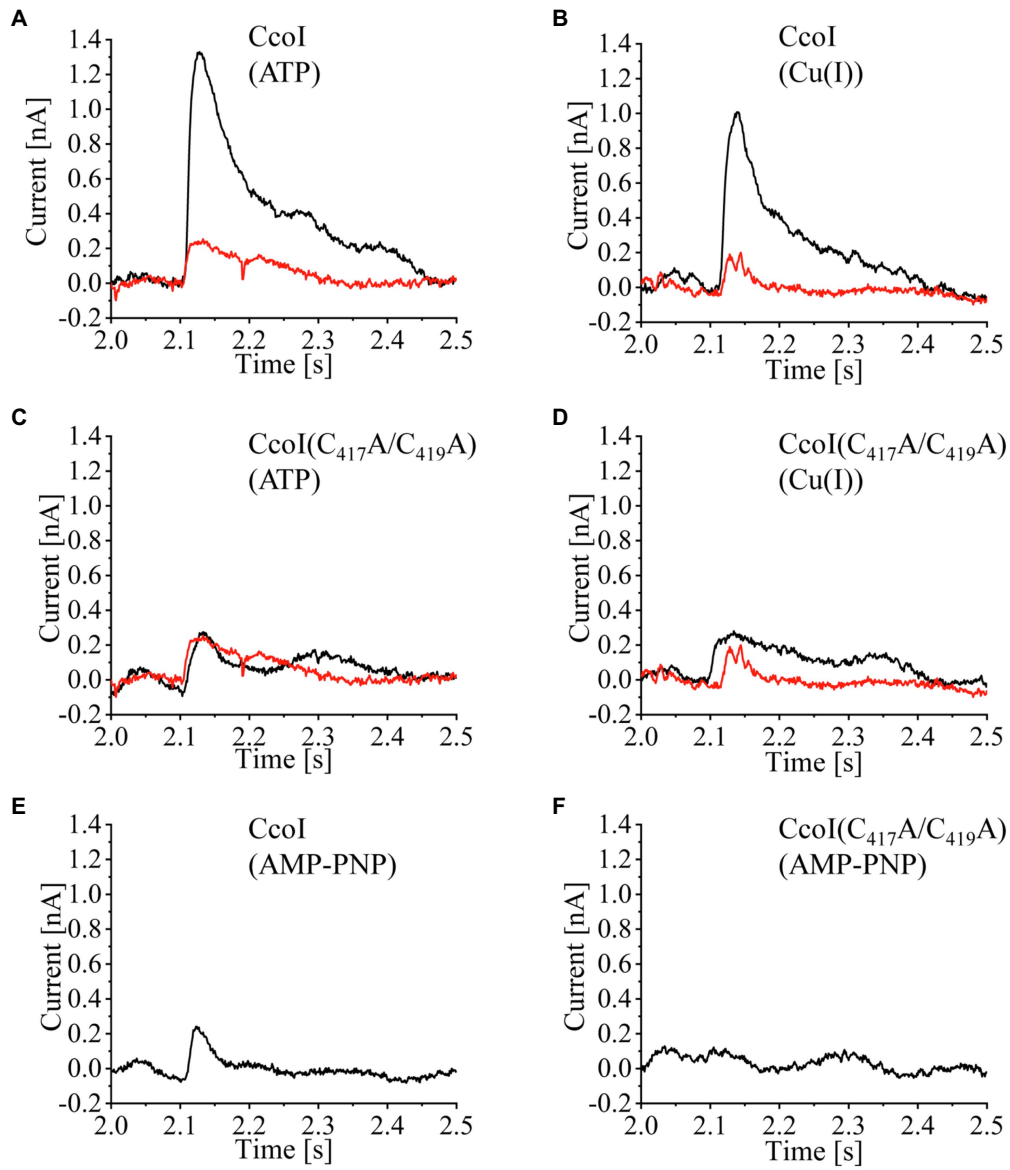
ATP-dependent Cu(I) translocation by CcoI. Wild-type CcoI **(A,B,E)**, and the CcoI(C_417_A/C_419_A) variant with a mutated TM-MBS1 **(C,D,F)**, were reconstituted at a 5:1 LPR (lipid-to-protein ratio) in liposomes (LP) prepared using a mixture of DSPC(1,2-distearoyl-sn-glycero-3-phospho-choline), POPE (1,2-dipalmitoleoyl-sn-glycero-3-phosphatidyl-ethanolamine), SOPG (1-stearoyl-2-oleoyl-sn-glycero-3-phospho-(1'-rac-glycerol)), and DGTS (1,2-dipalmitoyl-sn-glycero-3-O-4'-(N,N,N-trimethyl)-homoserine) at a 2:3.5:3.5:1 ratio. Representative current traces are shown. These traces were obtained when CcoI-LP (CcoI reconstituted in liposomes, black line) or LP (liposomes, red line) were adsorbed to a sensor unit and subsequently exposed to concentration jumps of 250μM ATP in the presence of 300μM Cu(I) **(A,C)**. In addition, traces of concentration jumps of 300μM Cu(I) in the presence of 250μM ATP were recorded **(B,D)**. **(E)** Same setup as in **(A)**, except that 250μM of the non-hydrolysable ATP analog AMP-PNP was used. **(F)** As in **(E)**, but the CcoI(C_417_A/C_419_A) variant was analyzed. All signals were recorded using a SURFE^2^R-N1 (Nanion Technologies, Munich, Germany).

The best experimental conditions were obtained when the vesicles were prepared using pure lipids (Supplementary Figures S3A, S4) and the non-activating (NA)-solution was composed of 25mM Tris-HCl, pH 7.4, 300mM KCl, 5mM MgCl_2_, 10mM ascorbic acid and 20mM cysteine (plus 300μM CuCl_2_ or 250μM ATP, as indicated). To investigate the effect of ATP in the presence of Cu(I), both NA- and activating (A)-solutions contained Cu, while ATP was only present in the A-solution. Likewise, to probe the effect of Cu in the presence of ATP, both solutions contained ATP, but only the A-solution had Cu. Under these conditions, when CcoI-LPs were subject to a concentration jump of ATP (250μM) in the presence of Cu (300μM), a transient current with an average amplitude of 1.3nA was recorded (Figure 2A, black trace), in line with a Cu(I) translocation event. In contrast, a control experiment using LPs subjected to the same cycle of solutions yielded a much smaller increase in amperage (Figure 2A, red trace), reflecting residual interactions of Cu(I) or ATP with the lipid environment. In the converse experiment, i.e., when CcoI-LP were flushed with A-solution containing 300μM Cu(I) in a background of 250μM ATP, similar current increases were also detected with only minor signals when LPs were used (Figure 2B). On average, the electrogenic signals observed for both the ATP-induced or Cu(I)-induced transient currents showed comparable amplitudes (Supplementary Figure S5A).

No significant responses were recorded when the CcoI(C_417_A/C_419_A) variant was reconstituted into LPs (Figures 2C,D). In this variant, alanine residues replaced the conserved cysteine residues from the Cu-binding TM-MBS1 motif. As expected, CcoI(C_417_A/C_419_A)-LPs did not show any electrogenic response upon activation by ATP (Figure 2C) or Cu(I) (Figure 2D). Similarly, when CcoI-LPs or CcoI(C_417_A/C_419_A)-LPs were activated with Cu(I) in the presence of the non-hydrolysable ATP analogue AMP-PNP, there was no electrogenic response, demonstrating that ATP hydrolysis is required for Cu(I) translocation by CcoI (Figures 2E,F). Additional controls included the activation of CcoI-LPs with ATP in a Cu(I) background containing the Cu(I)-chelator bathocuproinedisulfonic acid (BCS; 5mM) or in a background of 300μM Co(II) ions. In all these cases, the recorded transient currents were only residual (Supplementary Figures S5B, S5C).

Therefore, the overall data are consistent with an ATP-driven Cu(I) translocation by CcoI, and substantiate the earlier hypothesis that the absence of *cbb*_3_-Cox activity in the Δ*ccoI* strain is due to an impaired Cu-transport from the cytosol to the periplasm (Koch et al., 1998, 2000; Kulajta et al., 2006).

### High Cu(I) but Not Cu(II) Concentrations Inhibit ATPase Activity of CcoI

**Figure 3:**
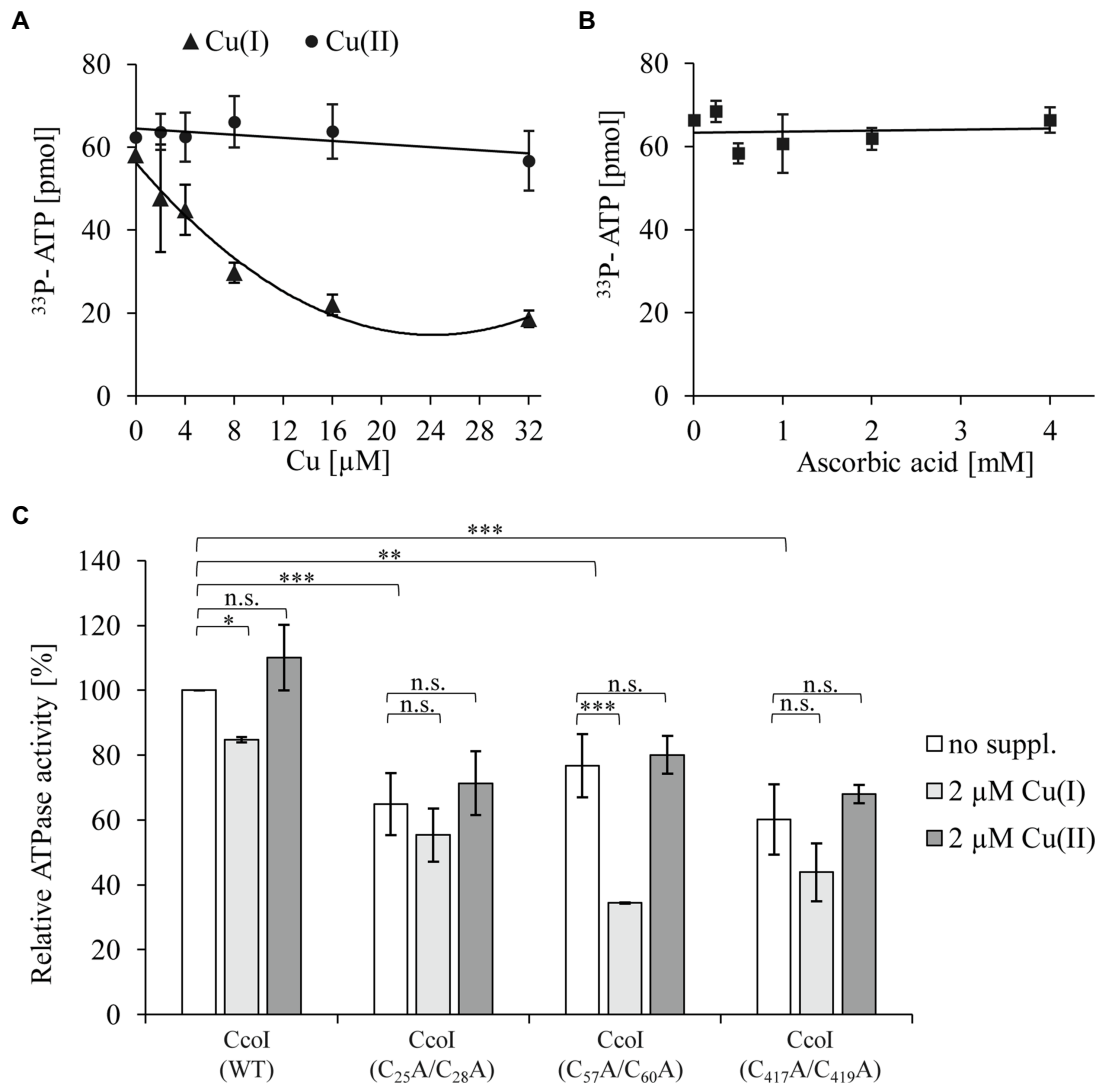
The ATPase activity of CcoI is inhibited by Cu(I) **(A)** The ATPase activity of CcoI was determined by quantifying the hydrolysis of ^33^P-γ-ATP in the presence of 1μM purified CcoI in detergent and at increasing concentrations of Cu(I) (▲, with ascorbic acid) or Cu(II) (⚫, without ascorbic acid). Released ^33^P was quantified *via* a scintillation counter. Error bars represent the standard deviation (SD) of three technical replicates. **(B)** As in A, but the ATPase activity of purified CcoI was analyzed in the presence of ascorbic acid concentrations (◼) equivalent to the ones used in **(A)** for Cu(I). **(C)** The influence of the different CcoI metal binding sites (MBSs) to the ATPase activity in the absence (no suppl., white bars) and presence of either 2μM Cu(I) (light grey bars) or 2μM Cu(II) (dark grey bars). The amount of hydrolyzed ATP by wild type CcoI in the absence of added Cu was set to 100% and the relative ^33^P release was calculated. Error bars represent the standard deviation (SD) of the mean values of three technical replicates. Statistical analyses were performed with the Satterthwaite corrected unpaired Students t-test. ^*^refers to values of *p*≤0.05; ^**^to values of *p*≤0.01; ^***^to values of *p*≤0.001 and (n.s.) to not significant.

The N-terminal metal binding site of CopA-like ATPases is suggested to serve as a Cu sensor that regulates the ATPase activity (Huster and Lutsenko, 2003; Mandal and Arguello, 2003; Wu et al., 2008). Considering the presence of two distinct N-MBSs in CcoI-like ATPases (Figure 1), the ATPase activity of detergent-solubilized, purified CcoI in the presence of different Cu(I) concentrations was analyzed by monitoring ^33^P-γ-ATP hydrolysis. The data revealed a Cu(I)-concentration-dependent decrease of the ATPase activity. Even at the lowest concentration tested (2μM), the ATPase activity was reduced by approx. 20% (Figure 3A). In contrast to Cu(I), the addition of Cu(II) up to a concentration of 32μM had no drastic effect on the ATPase activity of CcoI (Figure 3A). As Cu(I) was generated and maintained in its reduced state by the presence of ascorbic acid, the influence of equivalent ascorbic acid concentrations were also tested, but concentrations up to 4mM did not significantly influence the ATPase activity (Figure 3B).

Cu(I)-dependent inhibition of the ATPase activity was also observed for *Thermotoga maritima* CopA, but this required higher Cu(I) concentrations, and likely does not involve any MBSs (Hatori et al., 2008). The possible involvement of the CcoI-MBSs in Cu(I) mediated ATPase inhibition was analyzed by expressing three plasmid-borne *ccoI* mutant alleles in the Δ*ccoI* strain. The first CcoI variant contained alanine replacements in the glutaredoxin-like N-MBS1 [*ccoI(C_25_A/C_28_A*)], the second had similar replacements in the CopZ-like N-MBS2 [*ccoI(C_57_A/C_60_A*)], and the third was mutated in the TM-MBS1 [*ccoI(C_417_A/C_419_A*)], which was also used in the SSM experiments (Figure 2). These mutated CcoI variants were purified to homogeneity and their ATPase activity was analyzed.

The N-MBS1 mutation reduced the ATPase activity by approx. 30% (Figure 3C), compared to the wild type CcoI activity. This activity was further reduced by approx. 20% in the presence of Cu(I) (Figure 3C), similar to the inhibitory effect on wild type CcoI. The N-MBS2 mutation also reduced the ATPase activity by approx. 25%, compared to the wild type, but in this mutant, the addition of Cu(I) had a more pronounced inhibitory effect as it reduced the ATPase activity by 50%. The TM-MBS1 also showed an approx. 30% reduction of the ATPase activity, which dropped further in the presence of Cu(I). The inhibitory effect of Cu(I) on the TM-MBS1 mutant was comparable to that observed for wild type CcoI and the N-MBS1 mutant. As observed for the wild type (Figures 3A,C), the addition of 2μM Cu(II) did not significantly influence the ATPase activity in any of the CcoI mutants.

The ATPase activity of the TM-MBS1 mutant, which does not translocate Cu(I) based on the SSM experiments (Figure 2), indicates that ATP hydrolysis is not strictly coupled to Cu(I) binding or translocation. The data further show that the ATPase activity of CcoI is inhibited by already rather low Cu(I) concentrations, and that Cu(I) inhibition is more pronounced in the absence of N-MBS2. This finding suggests that, unlike CopA (Hatori et al., 2008), Cu(I) inhibition of CcoI activity is influenced by the MBSs, and that the ATPase activity of CcoI is particularly sensitive to Cu(I) in the absence of the N-MBS2. Note that the SSM experiments with CcoI-LPs were performed at Cu(I) concentrations that inhibited the ATPase activity of purified CcoI in detergent (Figures 2, 3). The lack of inhibition in the SSM experiments is probably explained by the presence of 20mM cysteine in the SSM buffers, which act as a strong Cu chelator and Cu donor (Pujol et al., 2011).

### Dissecting the Contributions of the CcoI Metal Binding Sites to *cbb_3_*-Cox Activity

**Figure 4:**
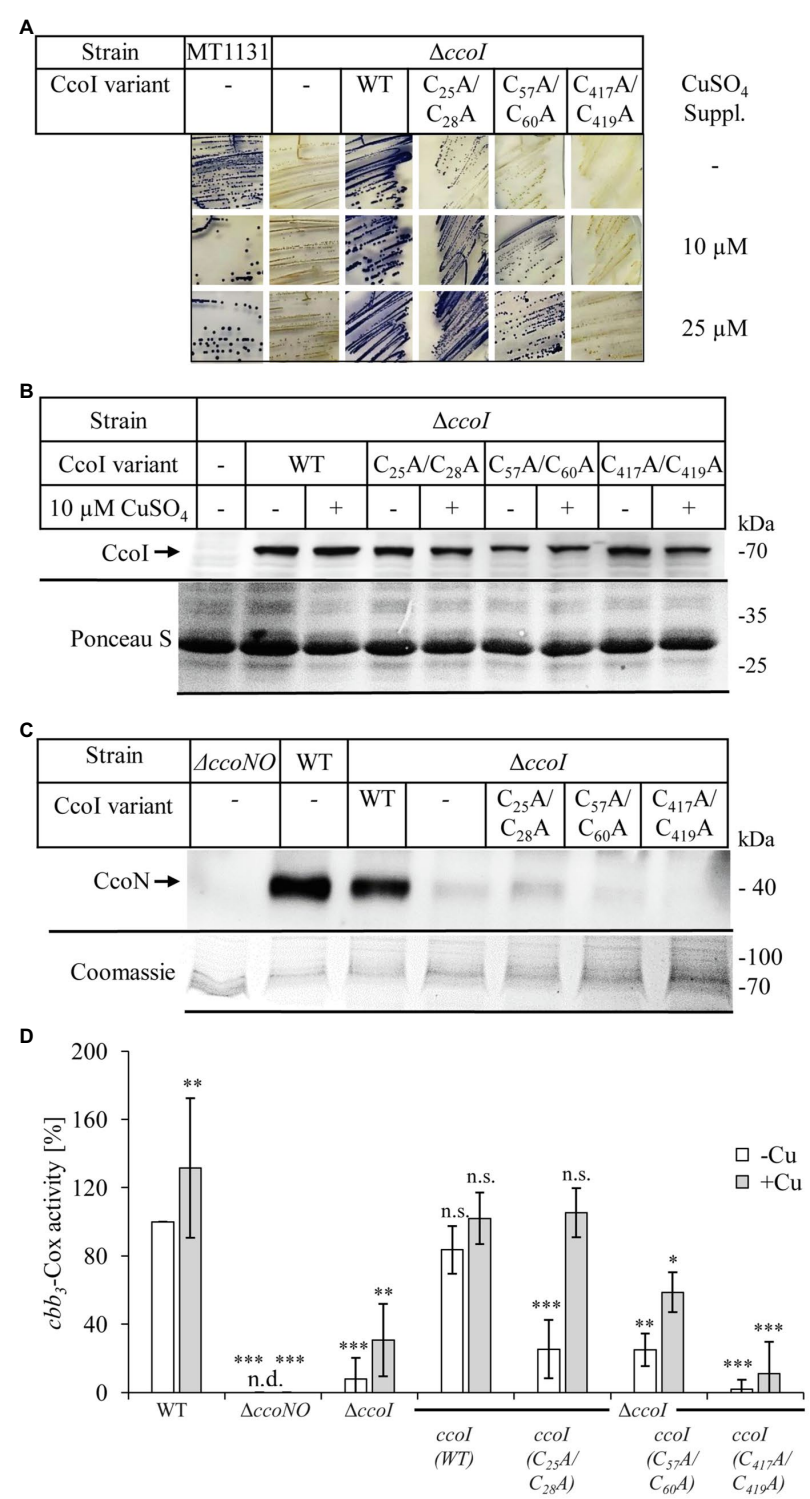
Distinct effects of the CcoI MBSs on *cbb_3_*-Cox activity. **(A)** NADI staining of wild type *R. capsulatus* MT1131 or the Δ*ccoI* strain, expressing different plasmid-encoded *ccoI* variants. The strains were grown semi-anaerobically on MPYE media, supplemented with 10μM, 25μM CuSO_4_ or without copper supplementation (−). 0.2% *L*-ara was added to the strains that carried a *ccoI* plasmid-borne copy to allow expression. Images showing the entire agar-plates are displayed in Supplementary Figure S6A. **(B)** 10^8^ cells of the indicated strains grown in the presence of 10μM CuSO_4_ or without copper supplementation were TCA precipitated, separated on a 12% SDS-PAGE and immune-blotted with antibodies against the Myc-tag (upper panel). As a loading control, the lower part of the same membrane was stained with Ponceau S (lower panel). Quantification of three biological replicates is shown in Supplementary Figure S6B. **(C)** Cytoplasmic membranes of the different *R. capsulatus* strains were isolated and 10μg of total protein were separated on a 16.5% SDS-Tris-Tricine polyacrylamide gel, immune-blotted and decorated with antibodies against CcoN (upper panel). As a loading control, the upper part of the gel was stained with Coomassie brilliant blue (lower panel). Quantification of three biological replicates is shown in Supplementary Figure S6C. **(D)** *cbb_3_*-Cox activity was determined *via* a DMPD oxidation assay. For each strain, 10^8^ cells grown to exponential phase were mixed with 1mM DMPD and monitored for 4min spectrophotometrically at 554nm. The WT activity was set to 100% and the relative activities of the other strains were calculated. The bars represent the mean values of three biological replicates with three technical repetitions each and the standard deviation is indicated by error bars (*n*=9). Statistical analyses were performed with the Satterthwaite corrected unpaired two-sided Student t-test, using the activity of the wild type grown in the absence of additional Cu as reference.^*^refers to values of *p*≤0.05; ^**^to values of *p*≤0.01, and ^***^to values of *p*≤0.001.

In the absence of CcoI, *cbb_3_*-Cox maturation is inhibited, resulting in very low amounts of the catalytic subunit CcoN and only background *cbb*_3_-Cox activities (Koch et al., 2000; Utz et al., 2019). This indicates that *cbb_3_*-Cox is a primary target of CcoI-mediated Cu(I) translocation. For dissecting further the contribution of the different MBSs in CcoI for *cbb_3_*-Cox activity in *R. capsulatus* cells, NADI-staining was performed as semi-quantitative assay for *cbb_3_*-Cox activity. In the presence of an active *cbb_3_*-Cox, α-naphthol and DMPD are converted to indophenol blue, which is detectable as blue color in *R. capsulatus* colonies (Koch et al., 1998; Figure 4A). Wild type MT1131 colonies turned blue within seconds (NADI^+^), while the Δ*ccoI* strain did not show any color reaction (i.e., NADI^−^), even on Cu-supplemented media (10μM or 25μM additional Cu; Figure 4A; Supplementary Figure S6A). Expressing a plasmid-borne copy of *ccoI* in the Δ*ccoI* strain restored the NADI reaction (i.e., *cbb_3_*-Cox activity).

The *ccoI(C_25_A/C_28_A)* allele containing the mutated N-MBS1 in the Δ*ccoI* strain, resulted in a NADI^slow^ phenotype on media without Cu supplementation, and in a NADI^+^ phenotype on Cu-supplemented media (Figure 4A). In contrast, the *ccoI(C_57_A/C_60_A;* N-MBS2 mutant) allele failed to restore *cbb*_3_-Cox activity on media without Cu supplementation (NADI^−^), but these cells showed a NADI^+^ phenotype on Cu-supplemented media (Figure 4A). The expression of the *ccoI(C_417_A/_419_A)* allele in the Δ*ccoI* strain did not support *cbb_3_*-Cox activity (i.e., NADI^−^), in agreement with its inability to transport Cu (Figure 2). Immune detection using α-Myc antibodies on whole cells expressing the different *ccoI* versions, revealed reduced amounts of all three mutated CcoI variants in comparison to wild type CcoI (Figure 4B; Supplementary Figure S6B), possibly indicating their reduced stability. Importantly, the addition of Cu did not significantly change the levels of wild type CcoI or its variants (Figure 4B; Supplementary Figure S6B). The NADI^−^ phenotype of Δ*ccoI* strains expressing the different *ccoI* alleles with mutated MBS was validated by monitoring the presence of the Cu-containing catalytic subunit CcoN of *cbb*_3_-Cox *via* immune detection. CcoN was readily detectable in wild type membranes, and basically absent in Δ*ccoI* membranes (Figure 4C). When wild type CcoI was expressed in the Δ*ccoI* strain, the CcoN levels became comparable to the wild type. However, upon expression of the N-MBS mutant alleles, only a weak CcoN band could be detected (Figure 4C; Supplementary Figure S6C), supporting the results from the NADI staining in the absence of additional Cu supplementation. CcoN was basically absent when the TM-MBS1 mutant allele was tested in the Δ*ccoI* strain.

The semi-quantitative analyses *via* NADI staining were confirmed by DMPD-oxidation based on quantitative photometric *cbb*_3_-Cox activity assays using whole cells (Figure 4D). The activity in wild type (MT1131) cells grown without additional Cu was set to 100% and this value increased by approx. 30% when cells were grown in the presence of additional 25μM Cu. No activity was observed in the *cbb*_3_-Cox mutant strain GK32 (*ΔccoNO*), which contains a chromosomal deletion of the *ccoN* and *ccoO* structural genes of *cbb*_3_-Cox (Koch et al., 1998). The Δ*ccoI* strain exhibited less than 10% of the wild type activity, which increased to approx. 30% when cells were grown in the presence of additional Cu (10μM Cu). This is different from the previous results based on O_2_-consumption assays using purified membranes, which showed no influence of Cu supplementation to *cbb*_3_-Cox activity in the Δ*ccoI* strain (Koch et al., 1998; Kulajta et al., 2006). Whether this is related to the different assay conditions or to *cbb*_3_-Cox independent DMPD oxidation was not further evaluated. Almost wild-type *cbb*_3_-Cox activity was detected in the Δ*ccoI* strain expressing the plasmid-borne *ccoI* copy. In the Δ*ccoI* strain expressing the *ccoI(C_25_A/C_28_A)* allele, approx. 25% of the wild type activity was detected, which increased to full wild type activity in Cu-supplemented (10μM Cu) media. *CcoI(C_57_A/C_60_A)* expressing cells also showed approx. 25% of wild type activity without Cu supplementation, but the addition of Cu only increased the activity to approx. 60% of the wild type. Finally, the *ccoI(C_417_A/_419_A)* allele only allowed for 2% of the wild type activity, which was increased to approx. 10% when cells were grown in the presence of additional Cu (Figure 4D).

Overall data indicated that the function of the different MBSs in CcoI is dependent on external Cu availability. While the inactivation of the glutaredoxin-like N-MBS1 can be fully restored by Cu supplementation, the CopZ-like N-MBS2 yielded an intermediate phenotype that could only be rescued partially by Cu addition. Considering that the CopZ-like N-MBS2 is conserved between CcoI and CopA, while the presence of the N-MBS1 is a particular feature of the high-affinity CcoI, these findings suggested that N-MBS1 might be important for receiving Cu from an alternative Cu donor different than CopZ, such as glutaredoxin or even glutathione (Maghool et al., 2020; Stewart et al., 2020).

### The N-Terminal Metal Binding Sites Are Important Under Low Intracellular Copper Concentrations

The partial or full complementation of *cbb*_3_-Cox activity in the N-MBS mutants by Cu supplementation of the medium is likely due to increased intracellular Cu concentrations, which might exceed those usually observed in bacteria (Heldal et al., 1985; Rae et al., 1999; Ekici et al., 2014). Recent data indicated that deleting CopZ or CopA also increases the intracellular Cu concentrations (Utz et al., 2019). Thus, whether or not the two CcoI N-MBSs mutants could support *cbb_3_*-Cox assembly in the absence of CopZ or CopA was analyzed.

**Figure 5:**
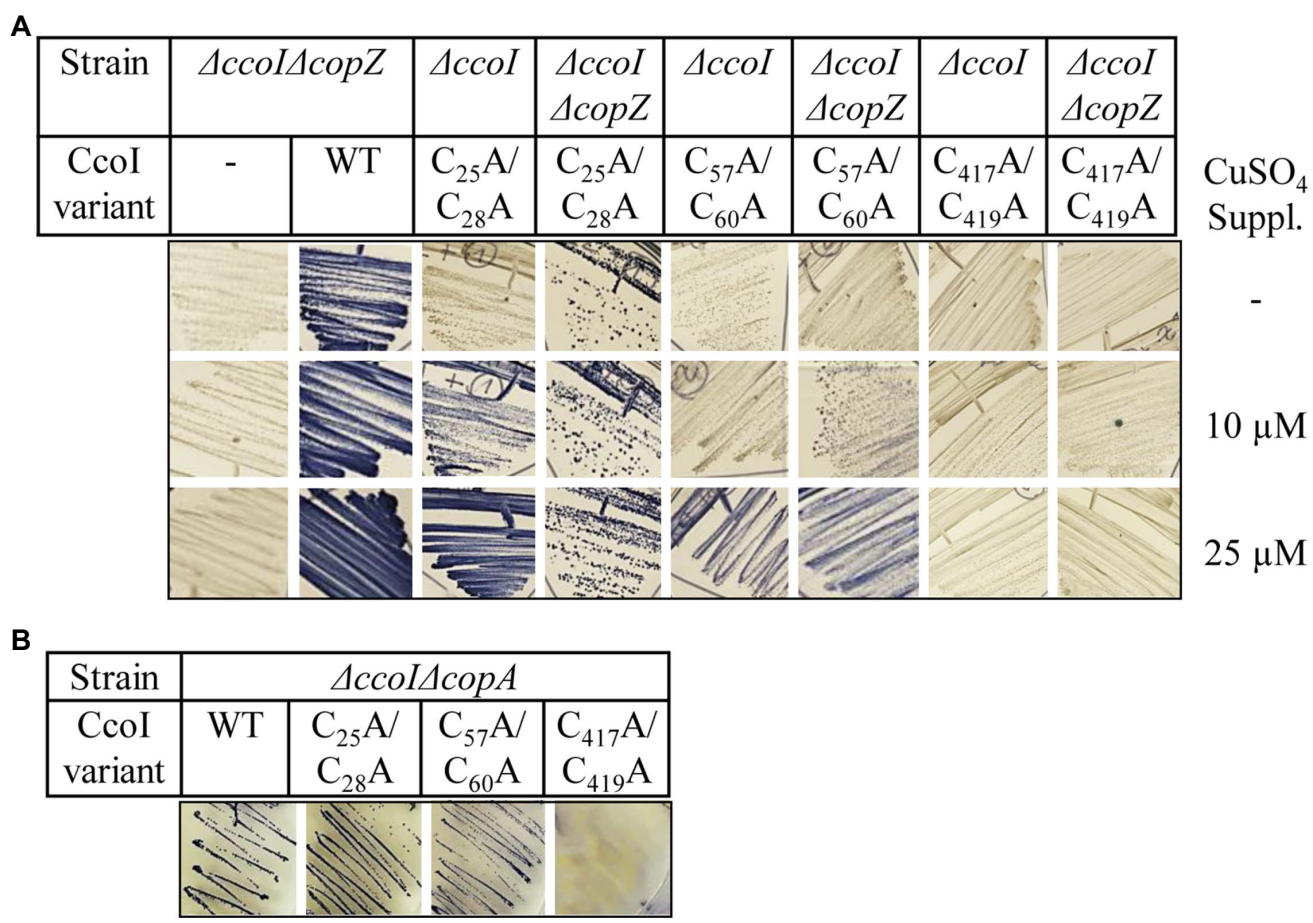
The cytoplasmic copper concentration influences metal acquisition by CcoI. **(A)** NADI staining of different *R. capsulatus* strains expressing either wild type *ccoI* from a plasmid (WT) or the indicated variants. Cells were grown on MPYE plates in the presence of 10μM or 25μM CuSO_4_ or without copper supplementation. **(B)** NADI staining of the *R. capsulatus* Δ*ccoI*Δ*copA* double-knock-out expressing different *ccoI* alleles. Images showing the entire agar-plates are displayed in Supplementary Figure S7.

The CcoI variants were conjugated into a Δ*ccoI*Δ*copZ* strain and the cells were grown on MPYE medium without Cu supplementation and with 10μM or 25μM CuSO_4_ addition. The Δ*ccoI*Δ*copZ* strain showed a NADI^−^ phenotype independent of Cu supplementation (Figure 5A) and was rescued to NADI^+^ phenotype with a plasmid-borne copy of *ccoI*, supporting the earlier observation that CopZ is involved in, but not essential for *cbb_3_*-Cox assembly (Utz et al., 2019). This is also confirmed by the NADI^+^ phenotype of the Δ*copZ* single mutant (Utz et al., 2019). When the *ccoI(C_25_A/C_28_A)* or the *ccoI(C_57_A/C_60_A)* alleles were expressed in the Δ*ccoI* strain, the external Cu-dependent *cbb_3_*-Cox activities were observed as before (Figures 4A, 5A). However, the expression of the *ccoI(C_25_A/C_28_A)* allele in the Δ*ccoI*Δ*copZ* double mutant allowed for a weak NADI^+^ even in the absence of additional Cu (Figure 5A). The *ccoI(C_57_A/C_60_A)* allele started to restore the NADI phenotype of the Δ*ccoI*Δ*copZ* double mutant strain at 10μM CuSO_4_ or above (Figure 5A; Supplementary Figure S7). As a control, expression of the *ccoI(C_417_A/C_419_A)* allele did not rescue the *cbb*_3_-Cox activity of the Δ*ccoI*Δ*copZ* strain, even in the presence of Cu supplementation, indicating that the rescue phenotypes observed were specific to the N-MBS mutants of CcoI.

When CopA is missing, the increase of intracellular Cu concentration is even more pronounced than that seen in the Δ*copZ* strain (Utz et al., 2019). When the CcoI N-MBS mutant alleles were expressed in the Δ*ccoI*Δ*copA* double mutant, both of these alleles were able to rescue *cbb_3_*-Cox activity even without any Cu supplementation (Figure 5B), while the TM-MBS1 mutant could not regain any *cbb_3_*-Cox activity (Figure 5B; Supplementary Figure S7).

In summary, these data indicate that increasing the intracellular Cu concentration due to the absence of the CopZ-CopA-mediated Cu export pathway is sufficient to compensate for the absence of the N-MBSs in CcoI, while the inactivation of TM-MBS1 cannot be bypassed by even large amounts of extracellular Cu supplementation. The different responses of the CcoI N-MBS1 and N-MBS2 mutants to external Cu addition both in the presence and absence of CopZ support the idea that the two N-MBSs have distinct roles in CcoI function.

### The CcoI-SenC Interaction Is Required For *cbb_3_*-Cox Activity

Due to the toxicity of free Cu, most cellular Cu is chelated by dedicated cytosolic and periplasmic Cu chaperones (Andrei et al., 2020), which transiently interact with P_1B_-type ATPases to deliver or receive Cu (Gonzalez-Guerrero et al., 2009; Fu et al., 2013; Padilla-Benavides et al., 2014). A transient interaction of CopZ with CcoI on the cytoplasmic side of the membrane was recently observed (Utz et al., 2019). However, whether CcoI also interacts with periplasmic chaperones, like SenC or PccA, remains unknown. For detecting possible contacts between CcoI and SenC or PccA, BN-PAGE analyses were performed, followed by immune detection using c-Myc antibodies. The data indicated that wild-type CcoI runs as a stable entity at approx. 150kDa (Supplementary Figure S8A), which would be consistent with a CcoI dimer. Dimerization of P_1B_-type ATPases has been observed before for ATP7B in humans and for CopA in *A. fulgidus* (Jayakanthan et al., 2017). However, when CcoI-containing membranes were solubilized with SDS (instead of DDM) prior to BN-PAGE, the 150kDa band remained intact (Supplementary Figure S8B), suggesting that it rather reflected a CcoI monomer. Determining the exact size of a protein complex *via* BN-PAGE is difficult and many proteins/protein complexes show aberrant migrations on BN-PAGE due to the binding of DDM and Coomassie (Heuberger et al., 2002; Kulajta et al., 2006). The 150kDa band was also observed in membranes of the Δ*ccoI* strain expressing the modified MBS alleles of CcoI (Supplementary Figure S8A) while it was absent in Δ*ccoI* membranes. Upon Cu supplementation, additional weaker bands became visible in all strains at 250kDa, 400 KDa, and 600kDa (Supplementary Figure S8A). However, these entities were also visible in the Δ*senC* and Δ*pccA* strains and antibodies against SenC or PccA do not recognize any of them (data not shown), suggesting that SenC and PccA are not present in these complexes.

**Figure 6:**
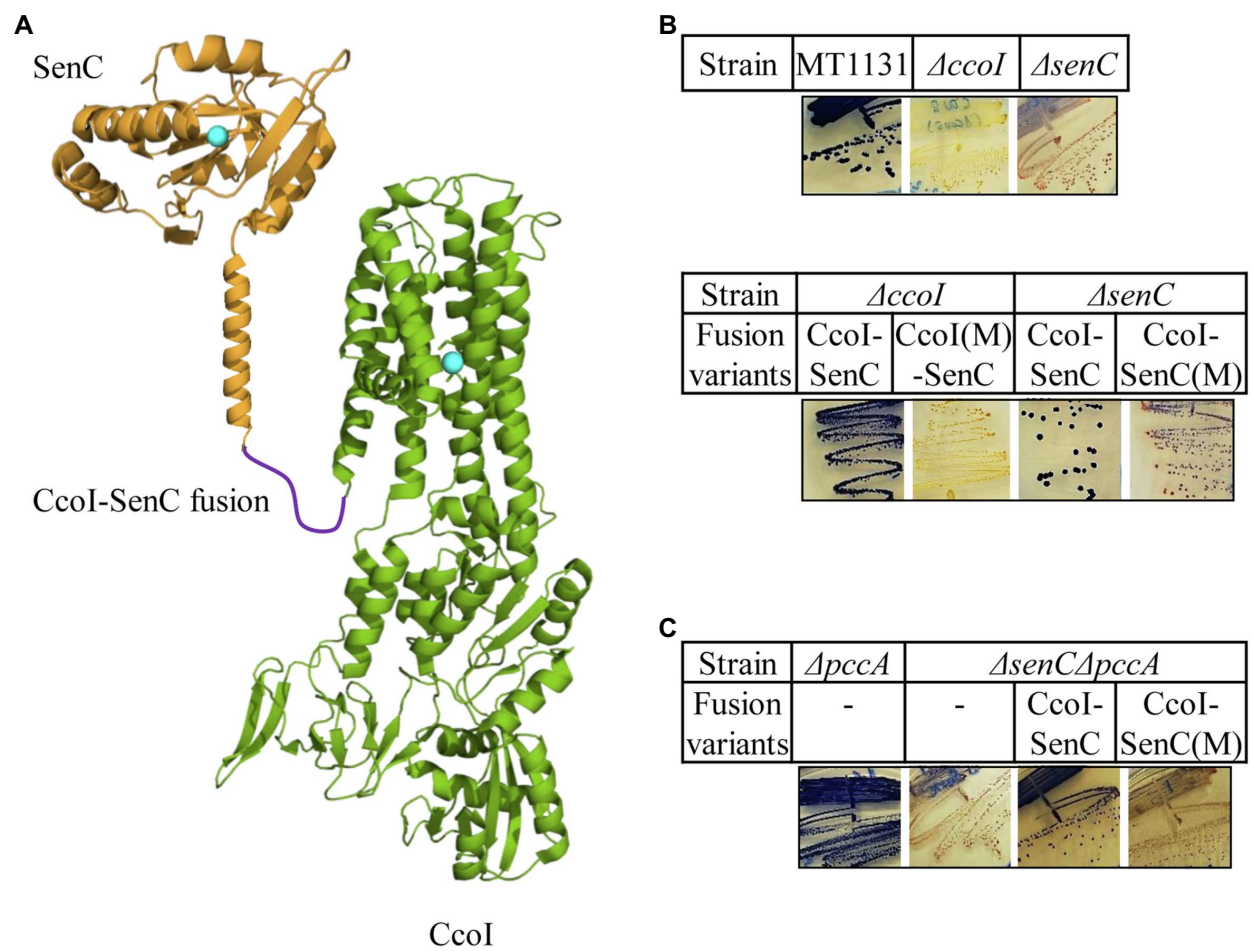
A CcoI-SenC fusion protein is sufficient for Cu delivery to *cbb_3_*-Cox. **(A)** Cartoon showing the genetically fused CcoI-SenC fusion protein, using known structures of the SenC-homologue ScoI (PDB 4WBR) and of CopA (PDB 3RFU). The Cu binding site in SenC and the TM-MBS1 of CcoI are indicated by turquoise spheres. **(B)** NADI staining of the wild type MT1131 and the Δ*ccoI* and Δ*senC* single mutants (upper panel), and the Δ*ccoI* or Δ*senC* single mutants expressing different CcoI-SenC fusion proteins (lower panel) CcoI(M)-SenC designates a fusion protein in which the fusion protein contains the *ccoI*(C_417_A/C_419_A) mutation in TM-MBS1. CcoI-SenC(M) designates a fusion protein in which the Cu binding site of SenC is mutated (*senC*(C_83_A/C_87_A) mutation). **(C)** NADI staining of the Δ*pccA* single and a Δ*pccA*Δ*senC* double knock-out strain expressing the CcoI-SenC fusion proteins when indicated.

Our earlier findings demonstrated that the periplasmic chaperone SenC is crucial for *cbb*_3_-Cox assembly at low Cu concentrations as its absence abolishes almost completely *cbb*_3_-Cox activity (Lohmeyer et al., 2012; Trasnea et al., 2016, 2018). SenC was also shown to directly interact with *cbb*_3_-Cox (Lohmeyer et al., 2012). In contrast, deleting PccA reduces *cbb*_3_-Cox activity only by 30% and the corresponding strain retains its NADI^+^ phenotype (Trasnea et al., 2016). We reasoned that if SenC serves as a periplasmic Cu acceptor for the CcoI-transported Cu, then a CcoI-SenC fusion protein should facilitate Cu transfer to *cbb*_3_-Cox. Such an approach has been employed before for monitoring electron-transfer reactions (Lee et al., 2008). A CcoI-SenC fusion protein was therefore constructed in which the C-terminus of CcoI was fused in-frame with the N-terminal TM of SenC. As controls, the TM-MBS1 of CcoI, and the Cu-binding site of SenC were mutated in two separate CcoI-SenC fusion constructs, generating the *ccoI(M)-senC and ccoI-senC(M)* alleles, respectively (Figure 6A). The wild type fusion construct and its mutant variants were expressed in the NADI^−^ Δ*ccoI* and Δ*senC* strains and their NADI phenotypes were monitored. The CcoI-SenC fusion protein was able to rescue both the Δ*ccoI* and Δ*senC* strains to NADI^+^ (Figure 6B), demonstrating that this entity is produced in an active form in these strains. Conversely, the *ccoI(M)-senC* allele failed to rescue the Δ*ccoI* strain, and similarly, the *ccoI-senC(M)* allele failed to rescue the Δ*senC* strain (Figure 6B), indicating that the CcoI-SenC fusion protein is fully functional only when the Cu binding sites in both proteins are intact. The functionality of the CcoI-SenC fusion furthermore supports their close interaction and the possibility of a direct Cu transfer from CcoI to SenC.

Although not essential for *cbb*_3_-Cox assembly, PccA can exchange Cu(I) with SenC, suggesting that it might also cooperate with SenC for Cu transfer to *cbb*_3_-Cox, at least under certain conditions. Thus, the CcoI-SenC fusion constructs were also tested in a Δ*senC*Δ*pccA* double mutant, which is NADI^−^ due to the absence of SenC. As expected, expression of the wild type CcoI-SenC fusion protein restored the NADI^+^ phenotype in the Δ*senC*Δ*pccA* strain, while that of the *ccoI-senC(M)* allele was unable to do so (Figure 6C), showing that the fusion protein was also active in a strain where a native CcoI was also present. In summary, these data suggest that a CcoI-SenC interaction is critical for *cbb*_3_-Cox assembly and that it likely naturally occurs in *R. capsulatus* membranes.

## Discussion

In the current work, the P_1B_-type ATPase CcoI of *R. capsulatus* was investigated. In contrast to its well-studied paralog CopA, which acts as the primary Cu exporter when cells are exposed to high Cu concentrations (Yang et al., 2007; Gonzalez-Guerrero and Arguello, 2008; Ekici et al., 2014; Mattle et al., 2015; Purohit et al., 2018; Utz et al., 2019; Abeyrathna et al., 2020), a detailed characterization of CcoI was hitherto lacking. Previous studies had demonstrated that CcoI is essential for the assembly and activity of *cbb*_3_-Cox in bacteria, including *R. capsulatus* (Koch et al., 2000; Kulajta et al., 2006; Utz et al., 2019), *Bradyrhizobium japonicum* (Preisig et al., 1996) and *Rubrivivax gelatinosus* (Hassani et al., 2010). Based on the high sequence conservation between CopA and CcoI (33.2% sequence identity; Supplementary Figure S1), including its putative metal-specificity conferring amino acids (Ekici et al., 2014), it was proposed that CcoI could export Cu for the assembly of the heme *b*-Cu_B_ binuclear center in CcoN, the catalytic subunit of *cbb*_3_-Cox (Kulajta et al., 2006). However, it was also found that the addition of external Cu could not rescue *cbb*_3_-Cox activity in the Δ*ccoI* strain (Koch et al., 1998, 2000; Kulajta et al., 2006). In contrast, in strains lacking the periplasmic Cu chaperones SenC or PccA, the *cbb*_3_-Cox activity can be rescued by increased external Cu concentrations (Lohmeyer et al., 2012; Trasnea et al., 2016, 2018). This observation is rather surprising because both chaperones are thought to transfer Cu from CcoI to *cbb*_3_-Cox during maturation (Lohmeyer et al., 2012; Trasnea et al., 2016). In addition, the *cbb*_3_-Cox defect in the Δ*ccoI* strain is also not remediated by increasing the cellular amounts of CopA, even though this was found to enhance Cu transport into the periplasm and to confer Cu resistance (Utz et al., 2019).

Despite sharing high sequence conservation with CopA and having an undisputed involvement in *cbb*_3_-Cox assembly, the ultimate proof that CcoI indeed transports Cu was lacking. For addressing this, we have produced and purified CcoI, reconstituted it into liposomes and performed solid-supported membrane (SSM)-based electrophysiology measurements, in analogy to what was done for CopA-like P_1B_-type ATPases (Abeyrathna et al., 2020). Our results revealed that the presence of CcoI is absolutely required to observe electrogenic transient currents with amplitudes well above background, and these can only be elicited by Cu(I) when ATP (but not a non-hydrolysable analog) is present. These results clearly establish that, like CopA, CcoI also functions as an ATP-dependent Cu exporter.

**Figure 7:**
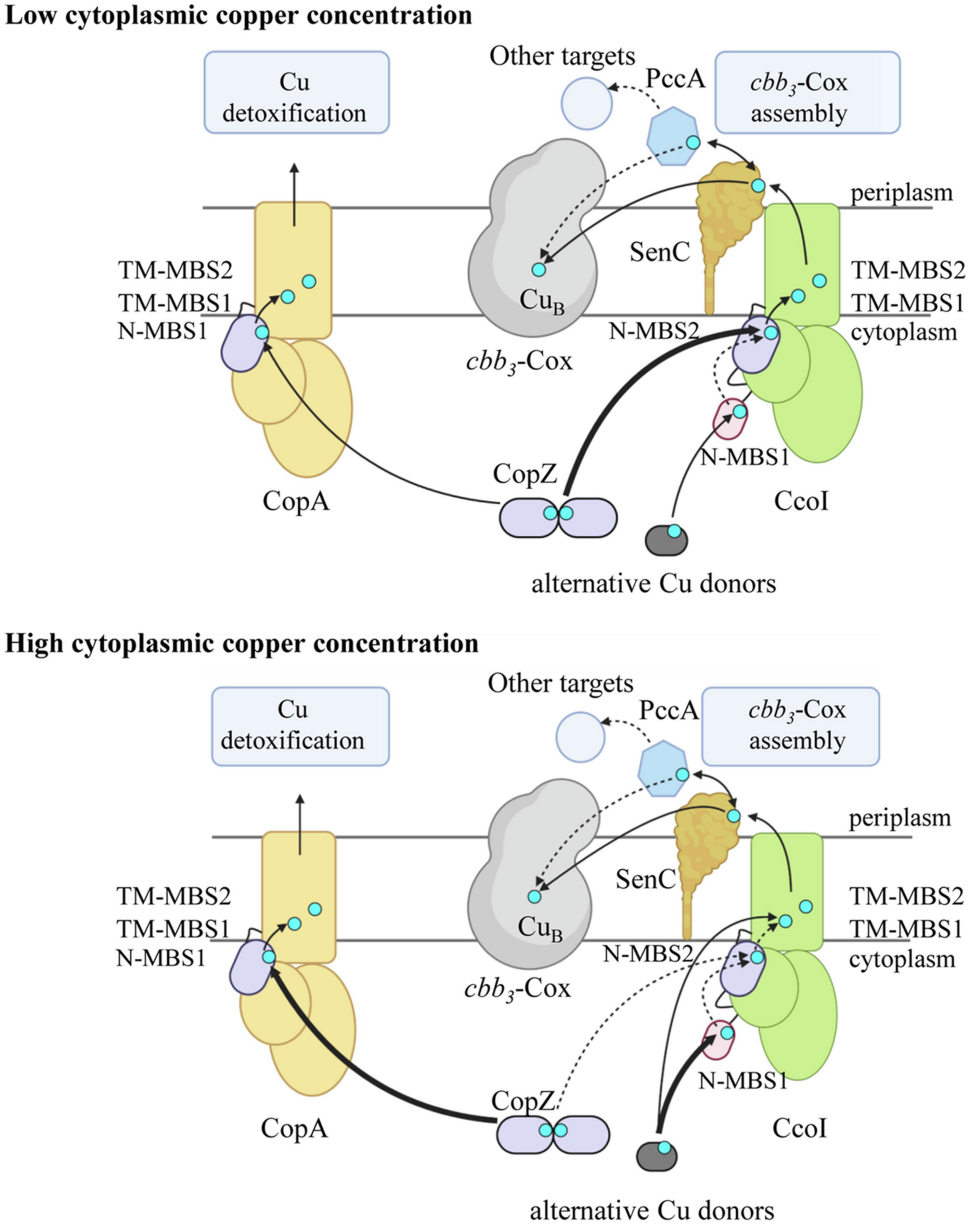
Model on Cu concentration-dependent Cu loading of CcoI in *R. capsulatus*. At low cytoplasmic Cu concentration, the CopA levels (light orange) are low and the N-terminal metal binding site N-MBS2 (purple) of CcoI (green) is loaded primarily by the cytoplasmic Cu-chaperone CopZ (purple; thick arrow). Cu loading of N-MBS2 is suggested to facilitate the Cu translocation reaction *via* the two transmembrane Cu binding sites (TM-MBS1 & TM-MBS2). The glutaredoxin-like N-MBS1 is likely accepting Cu from alternative Cu donors such as glutaredoxin or glutathione (dark gray) and Cu-loading of N-MBS1 is likely involved in regulating the ATPase activity of CcoI. Whether Cu can be transferred from N-MBS1 to N-MBS2 is not known (dotted arrow). From CcoI, Cu is transferred *via* the periplasmic Cu chaperone SenC (dark orange) to *cbb*_3_-Cox (light gray) for maturation of the heme *b*-Cu_B_ binuclear center in CcoN. SenC can also exchange Cu with the periplasmic Cu chaperone PccA (blue hexagon), which potentially could metalate other cuproproteins. At high cytoplasmic Cu concentrations, CopZ transfers Cu preferentially to CopA for Cu detoxification (thick arrow). As most CopZ is occupied with Cu delivery to CopA, Cu loading of CcoI is preferentially achieved by alternative Cu donors (thick arrow), likely *via* N-MBS1, but residual loading of N-MBS2 by CopZ can still occur (dotted arrow). In the absence of either N-MBS1 or N-MBS2 or CopZ, Cu translocation by CcoI can still occur, likely because the alternative Cu donors can deliver Cu also directly to TM-MBS1 for Cu translocation. At high Cu concentrations, SenC can be bypassed by an unknown mechanism (not shown). All Cu transfer pathways likely occur at all Cu concentrations, but the preferred use is defined by cytoplasmic Cu concentrations. The figure was prepared using biorender (*biorender.com*).

Next, the role of the two N-MBSs of CcoI was analyzed. Many bacterial CopA-like ATPases contain one or two N-MBSs, which are characterized by a CopZ-like (MxCxxC) Cu binding motif and a ferredoxin-like fold (Fan et al., 2001; Banci et al., 2002; Mandal and Arguello, 2003; Wu et al., 2008; Zhou et al., 2012; Drees et al., 2015). In some cases, these motifs are separated by only a few amino acids, like in *B. subtilis* (Singleton et al., 2008), while in *E. coli*, they are 34 amino acids apart, which probably favors their independent communication with the other CopA domains (Barry et al., 2010; Leshane et al., 2010; Drees et al., 2015). While the TM-MBSs are essential for Cu translocation (Gonzalez-Guerrero et al., 2008, 2009), the N-MBSs in bacterial CopA-like ATPases appear to be non-essential (Fan et al., 2001; Gonzalez-Guerrero and Arguello, 2008; Drees et al., 2015). Instead, they are proposed to regulate the ATPase activity in response to Cu availability (Wu et al., 2008; Arguello et al., 2016) and to support Cu-loading of the TM-MBSs (Figure 7; Drees et al., 2015; Maghool et al., 2020). Current models suggest that in the absence of Cu, the N-MBS is interacting with the N- and the A-domains of CopA, preventing either ATP binding or phosphorylation of the P-domain (Tsivkovskii et al., 2001; Wu et al., 2008; Gonzalez-Guerrero et al., 2009).

Remarkably, although the TM-MBSs are conserved between the CopA-like and CcoI-like ATPases, there are differences in the N-MBSs. In *Rhizobium meliloti* and *B. japonicum*, the CcoI-like ATPase FixI contains only one N-MBS (Preisig et al., 1996), which resembles the CopZ-like Cu binding motif, but lacks the Met residue upstream of the CxxC motif. In *R. capsulatus* and *R. sphaeroides*, CcoI contains two N-MBSs, separated by 28 amino acids. N-MBS1 contains a glutaredoxin-like CPAC motif (Ströher and Millar, 2012), while N-MBS2 contains a CVAC motif, which also lacks the typical upstream Met residue of CopZ-like motifs. The presence of a glutaredoxin-like motif in N-MBS1 and the absence of the typical Met residue in the CopZ-like N-MBS2, conceivably allows CcoI to receive Cu also from Cu donors other than CopZ (Figure 7). The absence of the Met residue in CopZ-like Cu binding motifs does not influence Cu binding, but it increases the flexibility of this motif (Palm-Espling et al., 2013), most likely by reducing interactions with other hydrophobic residues (Rosenzweig et al., 1999; Poger et al., 2005). The absence of the Met residue in the N-MBS2 of CcoI could potentially position the cysteine residues in a more flexible conformation, suitable to receive Cu from alternative Cu donors, including glutaredoxin and small molecules, like glutathione or cysteine (Figure 7; Helbig et al., 2008; Banci et al., 2010c; Singleton et al., 2010; Aliaga et al., 2016; Bhattacharjee et al., 2017; Dolgova et al., 2017). These molecules have been linked to Cu metabolism in other species (Li et al., 2004; Helbig et al., 2008) and have been shown to interact with P_1B_-type ATPases (Rosenzweig et al., 1999; Maghool et al., 2020). A CopZ independent Cu transfer to CcoI would also explain, why *cbb*_3_-Cox activity is reduced by only 30% in the absence of CopZ (Utz et al., 2019).

Our data show that the function of both N-MBSs and the TM-MBS in *R. capsulatus* CcoI can be functionally dissected. The mutated CcoI variants are produced at a comparable level (Figure 4B), but show distinct effects on *cbb*_3_-Cox activity. Mutating either N-MBS in CcoI reduces *cbb*_3_-Cox activity by about 75%, while mutating the TM-MBS1 reduces it completely (Figure 4D). This indicates that both of the N-MBSs are important but not essential for CcoI-mediated Cu translocation (Figure 7). However, *cbb*_3_-Cox activity in the N-MBS1 mutant can be fully restored by additional Cu, while the N-MBS2 mutant is only partially rescued. This is different for the TM-MBS1 mutant, which does not support *cbb*_3_-Cox assembly even at higher Cu concentrations. The observation that the *cbb*_3_-Cox related phenotype of the N-MBS2 mutant lies between the phenotypes observed for the N-MBS1 and TM-MBS1 mutants, suggests that the main function of N-MBS2 is to increase the local Cu concentration in close proximity to the TM-MBSs, while Cu-binding to N-MBS1 is additionally required for regulating the ATPase activity (Figure 7). Although further studies are needed to firmly establish a regulatory role of N-MBS1, this possibility is in line with the reduced ATPase activity of the N-MBS1 mutant and increased Cu(I) sensitivity when N-MBS2 is mutated. Due to the similarity between the N-MBS2 of CcoI and the Cu binding motif of CopZ, it appears likely that Cu is transferred from CopZ to N-MBS2, similarly as observed for CopA-like ATPases (Figure 7; Mandal and Arguello, 2003; Singleton and Le Brun, 2007; Rodriguez-Granillo and Wittung-Stafshede, 2008; Boal and Rosenzweig, 2009; Gonzalez-Guerrero et al., 2009; Banci et al., 2010b; Argüello et al., 2013). This is in line with the transient interaction between CcoI and CopZ that has been observed in *R. capsulatus* (Utz et al., 2019). Cu-loading of N-MBS2 by CopZ possibly enhances the subsequent Cu translocation step (Arguello et al., 2016), which would explain why the N-MBS2 mutant cannot be fully rescued by increased Cu concentrations. The partial rescue of the N-MBS2 mutant by increasing Cu concentrations (Figure 7) might be due to the presence of a conserved Cu entrance platform on the cytoplasmic side of TM2 in CcoI. In CopA, this site has been shown to serve as an additional contact site for direct Cu delivery to TM-MBSs (Gonzalez-Guerrero and Arguello, 2008).

Our data show that the *in vitro* ATPase activity of CcoI is inhibited by rather low Cu(I), but not Cu(II), concentrations. At Cu concentrations of 5–40μM, which are often used for stimulating CopA-like ATPases (Drees et al., 2015), the CcoI-like ATPase is almost completely inhibited. CopA-like ATPases can also be inhibited by high Cu(I) concentrations, but this does not depend on the MBSs (Hatori et al., 2008), and rather appears to be unspecific because it has also been observed for Zn^2+^/Cd^2+^- transporting P-type ATPases (Schurig-Briccio and Gennis, 2012). This inhibition is different in the case of CcoI, as our data show that inactivation of N-MBS2 significantly increases Cu(I) sensitivity, while inactivating N-MBS1 does not influence Cu(I) inhibition. In *R. capsulatus*, Cu(I) induced inhibition of CcoI is likely physiologically relevant for coordinating Cu export *via* either CopA or CcoI. CopA-like ATPases are transcriptionally up-regulated in response to 10μM Cu supplementation (Quintana et al., 2017; Utz et al., 2019), while the expression of CcoI is down-regulated (Utz et al., 2019). However, at lower Cu concentrations (5μM), there is no significant change in the CcoI protein levels (Selamoglu et al., 2020). Thus, Cu(I)-induced substrate inhibition of CcoI might fine-tune the Cu export pathways, as its inhibition would prevent an overload of the Cu-delivery pathway to *cbb*_3_-Cox, while simultaneously boosting Cu-detoxification *via* the CopZ-CopA pathway.

P_1B_-type ATPases transfer Cu from cytosolic to periplasmic Cu chaperones and thus provide the link between the cytosolic and periplasmic Cu homeostasis systems (Rensing and Grass, 2003; Argüello et al., 2013; Andrei et al., 2020). *R. capsulatus* contains two characterized periplasmic Cu chaperones, SenC and PccA (Lohmeyer et al., 2012; Trasnea et al., 2016), which are predicted to transfer Cu from CcoI to *cbb*_3_-Cox (Figure 7; Andrei et al., 2020). A SenC-PccA complex has been observed *in vivo* (Trasnea et al., 2016) and copper exchange between both proteins can occur *in vitro* (Trasnea et al., 2018). However, it was still unknown whether SenC or PccA was the primary acceptor of CcoI-mediated Cu translocation. Our study now revealed that a genetically fused CcoI-SenC complex is sufficient to support *cbb*_3_-Cox assembly, even in the absence of PccA. This is in line with data showing that a Δ*pccA* strain still exhibits 60% of wild type *cbb*_3_-Cox activity, while the activity is dropped to less than 10% in the Δ*senC* strain, which is also characterized by increased cytosolic Cu concentrations (Trasnea et al., 2016). However, both mutants are fully rescued by increasing the Cu concentration in the medium (Lohmeyer et al., 2012; Trasnea et al., 2016). SenC has been shown to directly interact with *cbb*_3_-Cox by chemical cross-linking (Lohmeyer et al., 2012), suggesting that SenC transfers Cu from CcoI to *cbb*_3_-Cox (Figure 7). Although we cannot exclude that the mobility of SenC’s periplasmic Cu-binding domain might be partially restricted in the CcoI-SenC fusion protein, the fusion protein is active as indicated by its ability to rescue both the Δ*ccoI* and Δ*senC* strains. A functional link between CcoI and SenC is also deduced from the observation that *senC* is transcriptionally upregulated at high Cu concentrations in the Δ*ccoI* strain (Lohmeyer et al., 2012).

The role of PccA in Cu transfer to *cbb*_3_-Cox is less clear; PccA could be involved in conveying Cu to some periplasmic cuproproteins, including SenC. Detailed studies on the assembly of the di-copper center (Cu_A_ center) in subunit II of the *aa*_3_-type cytochrome *c* oxidase of *Bradyrhizobium diazoefficiens* have recently demonstrated that the first Cu is provided by the SenC-homologue Sco1 and that the PccA-homologue PcuC is required for regenerating Cu-loaded Sco1 and for providing the second Cu ion for the assembly of the Cu_A_ center (Canonica et al., 2019a, b). The Cu_B_ center of *cbb*_3_-Cox contains only one Cu and our data suggest that Cu-loading of SenC likely occurs *via* CcoI. Yet, PccA could serve as a back-up strategy for Cu loading of SenC (Figure 7), as supported by the observation that the SenC-PccA complex is stabilized in the absence of CcoI (Trasnea et al., 2016).

In summary, our study provides further insight into the complexity of cuproprotein biogenesis by demonstrating the ATP-dependent Cu translocation by CcoI, by dissecting the different functions of its two distinct N-terminal metal binding sites (N-MBSs), and by showing that a CcoI-SenC fusion protein is fully functional in Cu delivery to *cbb*_3_-Cox (Figure 7). This physical proximity suggests that SenC likely serves as acceptor for Cu that is translocated by CcoI into the periplasm and destined for *cbb*_3_-Cox maturation. Future studies monitoring direct Cu transfer from CcoI *via* SenC to *cbb*_3_-Cox will hopefully further validate this pathway.

## Data Availability Statement

The original contributions presented in the study are included in the article/Supplementary Material, further inquiries can be directed to the corresponding author.

## Author Contributions

AA, MR, YÖ, FD, SA, and H-GK contributed to the design of the study, the acquisition, analysis, and interpretations of the data. AM, ND, FF, and JR contributed to the acquisition, analysis, and interpretations of the data. All authors contributed to the article and approved the submitted version.

## Funding

This work was supported by grants from the Deutsche Forschungsgemeinschaft (DFG; RTG 2202, Project-ID 278002225 to H-GK and SA; SFB1381 Project-ID 403222702 to H-GK), and the Else-Kröner Fresenius Stiftung/Motivate MD college of the University Freiburg Medical School to JR. FD was supported by grants from NIH GM 38237 and the Division of Chemical Sciences, Geosciences, and Biosciences, Office of Basic Energy Sciences of Department of Energy [DOE DE-FG02-91ER20052]. MR was supported in part by the Excellence Initiative of the German Research Foundation (GSC-4, Spemann Graduate School) and in part by the Ministry for Science, Research and Arts of the State of Baden-Wuerttemberg. YÖ gratefully acknowledges support by Philipp-Schwartz-Initiative of the Alexander von Humboldt Foundation.

## Conflict of Interest

The authors declare that the research was conducted in the absence of any commercial or financial relationships that could be construed as a potential conflict of interest.

## Publisher’s Note

All claims expressed in this article are solely those of the authors and do not necessarily represent those of their affiliated organizations, or those of the publisher, the editors and the reviewers. Any product that may be evaluated in this article, or claim that may be made by its manufacturer, is not guaranteed or endorsed by the publisher.

## Footnotes

^1^ https://matheguru.com/
