# Supplementary material for: The CopA2-Type P1B-Type ATPase CcoI Serves as Central Hub for cbb3-Type Cytochrome Oxidase Biogenesis
Source: Front Microbiol. 2021 Sep 13;12:712465. doi: 10.3389/fmicb.2021.712465 (PMC8475189; doi:10.3389/fmicb.2021.712465)
Supplement: Supplementary file 1 [file Data_Sheet_1.PDF]

Table 1: Bacterial strains used in the present study

Strain	Genotype	Reference
E. coli		
C43(DE3)	F⁻ *omp*T *hsd*SB (r_B^- m_B^-) *gal dcm* (DE3)	(Miroux and Walker, 1996)
DH5-α	*sup*E44 D*lac*U169(F80*lac*ZDM15) *hsd*R17 *rec*A1 *end*A1 *gyr*A96 *thi*-1 *rel*A1	(Sambrook and Russel, 2001)
HB101	F⁻ Δ(*gpt-pro*A)62 *leu*B6 *sup*E44 *ara*14 *gal*K2 *lac*Y1 Δ (*mcr*C-*mrr*) *rps*L20 (*Str*R) *xyl*-5 *mtl*-1 *recA*13	(Sambrook and Russel, 2001)
R. capsulatus		
MT1131	crtD121 RifR ccoP269	(Dierstein, 1984)
Δ*ccoI* (CW2)	Δ*ccoI* ::*spe*	(Koch et al., 2000)
Δ*copZ*	Δ*copZ*::*kan*	(Utz et al., 2019)
Δ*ccoI* Δ*copZ*	Δ*ccoI* ::*spe* Δ*(copZ*::*kan*)	This work
Δ*ccoI* Δ*copA*	Δ*ccoI* ::*spe* Δ*(copA*::*kan*)	(Öztürk et al., Submitted)
Δ*senC* (LS01)	Δ*senC*	(Swem et al., 2005)
Δ*pccA* (IT1)	Δ*pccA* ::*kan*	(Trasnea et al., 2016)
Δ*senC*Δ*pccA* (IT10)	Δ*senC* Δ(*pccA*::*kan*)	
Y262 (GTA-overproducer)		(Yen et al., 1979)
GK32 (Δ*ccoNO*)	Δ*ccoNO*::*kan*	(Koch et al., 2000)

Table 2: Plasmids used in the present study

Plasmids		
pBAD-*ccoI*-Myc/HisA	*ccoI* cloned into pBAD-Myc/HisA via the NcoI/HindIII sites	(Utz et al., 2019)
pRK-*ccoI*	pBAD-*ccoI*-Myc/HisA cloned into pRK415 via the BamHI generated sites	
pRK2013	Conjugation helper plasmid (Km^r)	(Ditta et al., 1985)
pRK415	Broad host range vector (Tet^r)	
pBAD-*ccoI*($C_{25}A/C_{28}A$)-Myc/His	*ccoI* cloned into pBAD-Myc/HisA, in which alanins substitute cysteines 25 and 28	This work
pBAD-*ccoI*($C_{57}A,C_{60}A$)-Myc/His	*ccoI* cloned into pBAD-Myc/HisA, in which alanins substitute cysteines 57 and 60	
pBAD-*ccoI* ($C_{417}A,C_{418}A$)-Myc/His	*ccoI* cloned into pBAD-Myc/HisA, in which alanins substitute cysteines 417 and 419	
pRK-*ccoI* ($C_{25}A/C_{28}A$)	*ccoI* in pBAD/Myc/HisA with cysteines 25 and 28 substituted to alanins cloned into pRK415 via the BamHI generated sites	
pRK-*ccoI* ($C_{57}A/C_{60}A$)	*ccoI* in pBAD/Myc/HisA with cysteines 25 and 28 substituted to alanins cloned into pRK415 via the BamHI generated sites	
pRK-*ccoI* ($C_{417}A/C_{418}A$)	*ccoI* in pBAD/Myc/HisA with cysteines 25 and 28 substituted to alanins cloned into pRK415 via the BamHI generated sites	
pRK415-Δ*copZ*	Δ(*copZ*::*Gm*)	(Utz et al., 2019)
pRK-*CopA2*::Kan	Δ(*copA*::*kan*)	(Ekici et al., 2014)
pRK-*ccoI*-*senC*	pBAD-*ccoI*-*senC*-Myc/HisA cloned into pRK415 via the BamHI generated sites	This work
pRK-*ccoI*($C_{417}A/C_{418}A$)-*senC*	pBAD-*ccoI*-*senC*-Myc/HisA cloned into pRK415 via the BamHI generated sites. CcoI cysteines 417 and 419 were substituted to alanins.	
pRK-*ccoI*-*senC*($C_{83}A/C_{87}A$)	pBAD-*ccoI*-*senC*-Myc/HisA cloned into pRK415 via the BamHI generated sites. SenC cysteines 83 and 87 were substituted to alanins.	

Table 3: Primers used in the present study

Target gene/ plasmid	Primer	Primer sequence (5’→ 3’)
ccoI	1a	CGCTTCCGCCGCCCCGGCCGCCCTGGCCGCCCCCTC
	1b	GAGGGGGCGGCCAGGGCGGCCGGGGCGGCGGAAGCG
	2a	CACCGCCCATGCCGCGGTCGCCATCACCGATGTC
	2b	GACATCGGTGATGGCGACCGCGGCATGGGCGGTG
	3a	GATCATCACCGCTCCCGCCGCGCTGGGGCTG
	3b	CAGCCCCAGCGCGGCGGGAGCGGTGATGATC
pBAD -Myc/ HisA	4a	GATCCTCTAGAGTCGACCTGCACGAGGAAGCGGAAGAGCGCCTG
	4b	CGCCAAGCTTGCATGCCTGCACTCACTGACTCGCTGCGCTCG
	5a	GAACAAAAACTCATCTCAGAAGAGG
	5b	CATGGTTAATTCCTCCTGTTAGCC
ccoI	6a	CTTGTCATCGTCGTCCTTGTAGTCGCCCGAGCCGCCTTTCAGCCGCATC GAGTTCAGC
	6b	AACAGGAGGAATTAACCATGACCGCTGCCCCCCTTGCGTCG
senC	7a	ACAAGGACGACGATGACAAGGGCGGCTCGGGCGGCAACGTTTCGAGC AAGACCGCCG
	7b	TCTGAGATGAGTTTTTGTTCATTTCCGTTGCCCGCCGCGGG
ccoI	8a	TGCTGATCATCACCGCGCCCGCGGCGCTGGGGCTGGCCGTGCCTGC
	8b	GGGCGCGGTGATGATCAGCACGGCGGCC
senC	9a	ACAGCTATGCGCCCGACGTCGCGCCGATCGACAGCACCCGCAATGC
	9b	GACGTCGGGCGCATAGCTGTAACCGAAGTAGAC

References

Dierstein, R. (1984). Synthesis of pigment-binding protein in toluene-treated Rhodopseudomonas capsulata and in cell-free systems. *European Journal of Biochemistry* 138**,** 509-518.

Ditta, G., Schmidhauser, T., Yakobson, E., Lu, P., Liang, X.W., Finlay, D.R., Guiney, D., and Helinski, D.R. (1985). Plasmids related to the broad host range vector, pRK290, useful for gene cloning and for monitoring gene expression. *Plasmid* 13**,** 149-153.

Ekici, S., Turkarslan, S., Pawlik, G., Dancis, A., Baliga, N.S., Koch, H.-G., and Daldal, F. (2014). Intracytoplasmic copper homeostasis controls cytochrome c oxidase production. *mBio* 5**,** e01055-01013.

Koch, H.G., Winterstein, C., Saribas, A.S., Alben, J.O., and Daldal, F. (2000). Roles of the ccoGHIS gene products in the biogenesis of the cbb3-type cytochrome c oxidase. *Journal of Molecular Biology* 297**,** 49-65.

Miroux, B., and Walker, J.E. (1996). Over-production of proteins in Escherichia coli: mutant hosts that allow synthesis of some membrane proteins and globular proteins at high levels. *J Mol Biol* 260**,** 289-298.

Sambrook, J., and Russel, D.W. (2001). "Molecular Cloning". Cold Spring Harbor Laboratory).

Swem, D.L., Swem, L.R., Setterdahl, A., and Bauer, C.E. (2005). Involvement of SenC in assembly of cytochrome c oxidase in Rhodobacter capsulatus. *J Bacteriol* 187**,** 8081-8087.

Trasnea, P.I., Utz, M., Khalfaoui-Hassani, B., Lagies, S., Daldal, F., and Koch, H.G. (2016). Cooperation between two periplasmic copper chaperones is required for full activity of the cbb3 -type cytochrome c oxidase and copper homeostasis in Rhodobacter capsulatus. *Mol Microbiol* 100**,** 345-361.

Utz, M., Andrei, A., Milanov, M., Trasnea, P.I., Marckmann, D., Daldal, F., and Koch, H.G. (2019). The Cu chaperone CopZ is required for Cu homeostasis in Rhodobacter capsulatus and influences cytochrome cbb3 oxidase assembly. *Mol Microbiol* 111**,** 764-783.

Yen, H.C., Yhu, N.T., and Marrs, B.L. (1979). Characterization of the gene transfer agent made by an overproducer mutant of Rhodopseudomonas capsulata. *J. Mol. Biol.* 131**,** 157-168.

```
                                                  CcoI                      CcoI
                                                 N-MBS1                    N-MBS2
R. c. CcoI N-MBSs    MTAAPLASVAPPAGVTEDHLSASACPACLAAPSAEDLAKSGDIK--GARIMLSLPTAHCA    58
R. s. CcoI N-MBSs    MSLAER--I--DLPSSEAPGGASACPACLAAPSAERLAAMAGPA--GGRLVLSLPTAHCA    54
R. c. CopA N-MBS1    ----------------------------------------------MASTSFIVEGMHCG    14
R. s. CopA N-MBS1    ------------------------------------MTDQQPIRTGLRSLRLHPDGMSCA    24
                                                                ::      *.

R. c. CcoI N-MBSs    VCITDVEREMEAQPGVRSARVNLTLKRVSVDAEAGVSAETLVEALAKIGYEAYELDPG--    116
R. s. CcoI N-MBSs    ACMTTVEGGLEKLAGVRSARVNLTLRRVSVDAEPEVTAAQLVEALSRLGYEAHELDAG--    112
R. c. CopA N-MBS1    ACTGRVERALQAEAGVTLAAANLMARSVRVEFEAPARAETLAEALARAGYPIAEAETCLG    74
R. s. CopA N-MBS1    SCVARTERVLAAVPGVAEARVSLADESADIRYSDPATPEALAEALARAGYPARQERLTLS    84
                      *   .*  :    **  * ..*  . . :  .  .    *.***:: **   :
```

Figure S1: Sequence comparison of the N-terminal metal binding sites of CcoI (N-MBS1 and N-MBS2) with the N-terminal N-MBS1 of CopA. *R. capsulatus (R. c.)* and *R. sphaeroides (R. s.)*. Sequences were retrieved from Uniprot or from the KEGG databank. Numbers refer to the amino acid residues of the respective proteins. (*) indicate identical amino acids, (:) indicate amino acids with highly similar properties and (.) indicate amino acids with similar properties. Alignments were performed with Clustal Omega.

A.

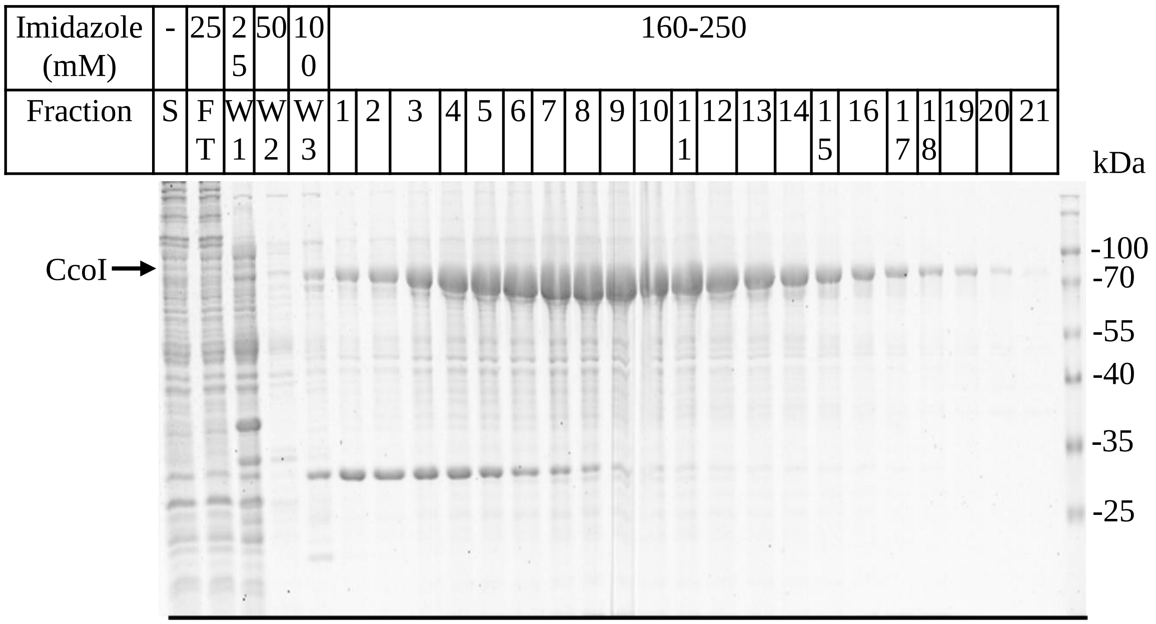

B.

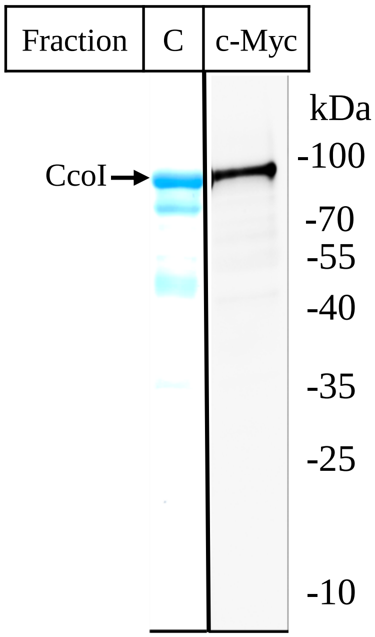

Figure S2: Purification of CcoI from *E. coli* membranes**.** (**A**) CcoI was heterlogousely expressed in *E. coli* and purified as described in Material and Methods. The dodecyl-maltoside-solubilized membrane fraction (S) was loaded onto a HisTrap HP affinity column and unbound proteins are present in the flow-through fraction (FT). Increments in the imidazole concentration allowed to selectively elute protein contaminants (W1-W3) from the column. The individual elution fractions of the imidazole gradient are shown. 100 µL samples were precipitated with 5% TCA, separated on 12% SDS-PAGE and stained using a Coomassie-Blue solution. (**B**) All CcoI eluted fractions were subsequently combined and concentrated. The concentrated CcoI fraction from (A) was loaded onto a S200 26/60 size-exclusion column and 15 µL of the monomeric CcoI-containing fraction were applied on 12% SDS-PAGE and Coomassie-Blue stained (C) or immuno-blotted with antibodies against the c-Myc tag.

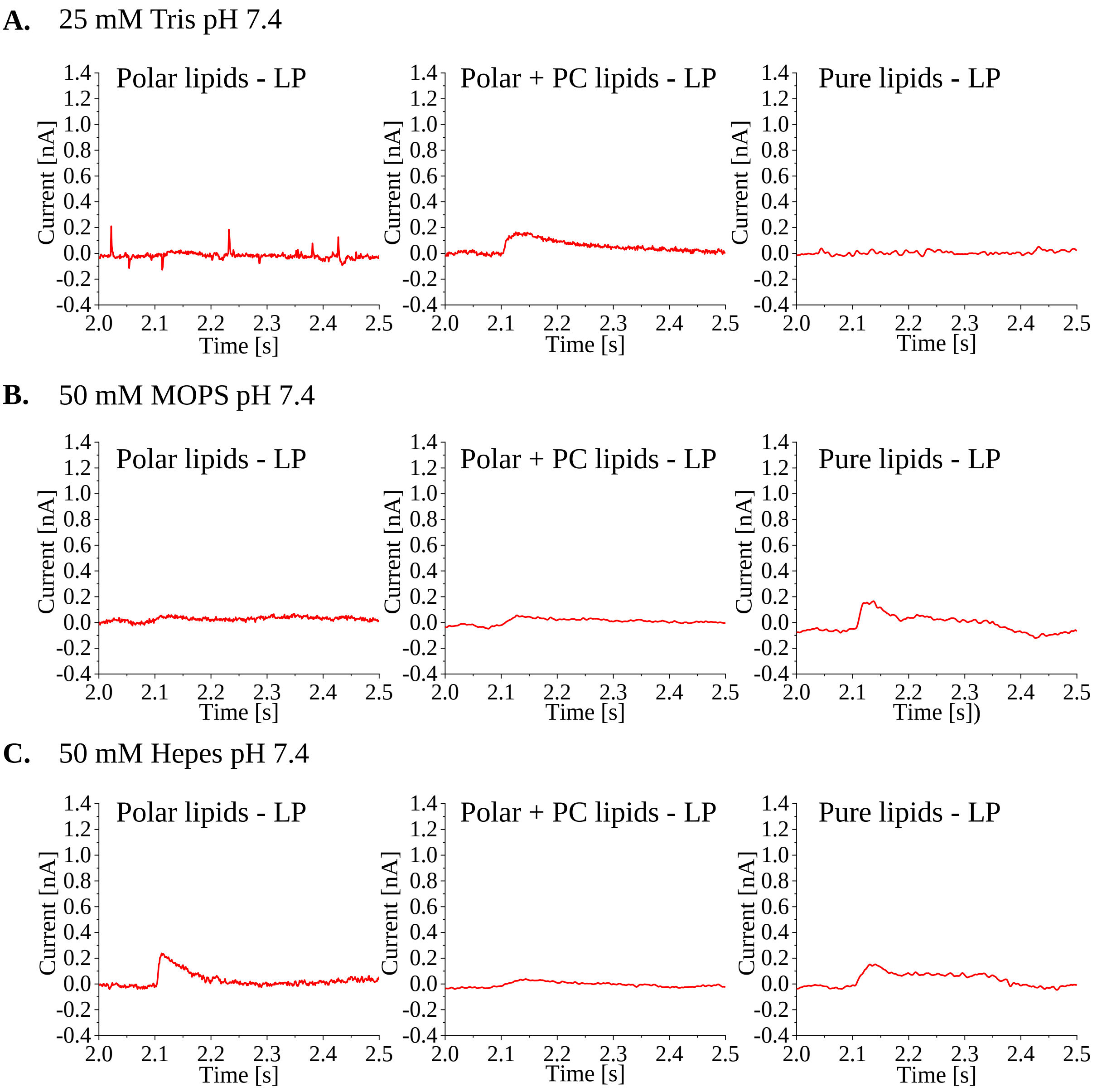

Figure S3: Optimization of the experimental conditions to perform solid-supported membrane electrophysiology. In the absence of protein, electrogenic events arising from solution exchange artifacts, and in particular from the undesired interaction of ions with lipids should be minimized. Here, liposomes (LP) prepared using three different lipid mixtures (*E. coli* polar lipids (Avanti Polar Lipids), a 3:2 suspension of *E. coli* polar lipids and phosphatidylcholine (PC) and a 2:3.5:3.5:1 suspension of DSPC, POPE, SOPG and DGTS) were adsorbed onto sensor units, placed inside a SURFE^{2}R-N1 Faraday cage and subject to cycles of non-activating (NA-)/activating (A-)/NA-solution perfusions as described in the text. Representative transient currents are depicted for situations where distinct liposomes were activated by concentration jumps in ATP (250 µM) on a Cu(I) background (500 µM). In the cases shown, both A- and NA-solutions contained 300 mM KCl, 5 mM $MgCl_2$, 10 mM ascorbic acid, 20 mM cysteine and 500 µM $CuCl_2$ and in (**A**) 25 mM Tris pH 7.4, in (**B**) 50 mM MOPS pH 7.4 or in (**C**) 50 mM Hepes pH 7.4 buffer.

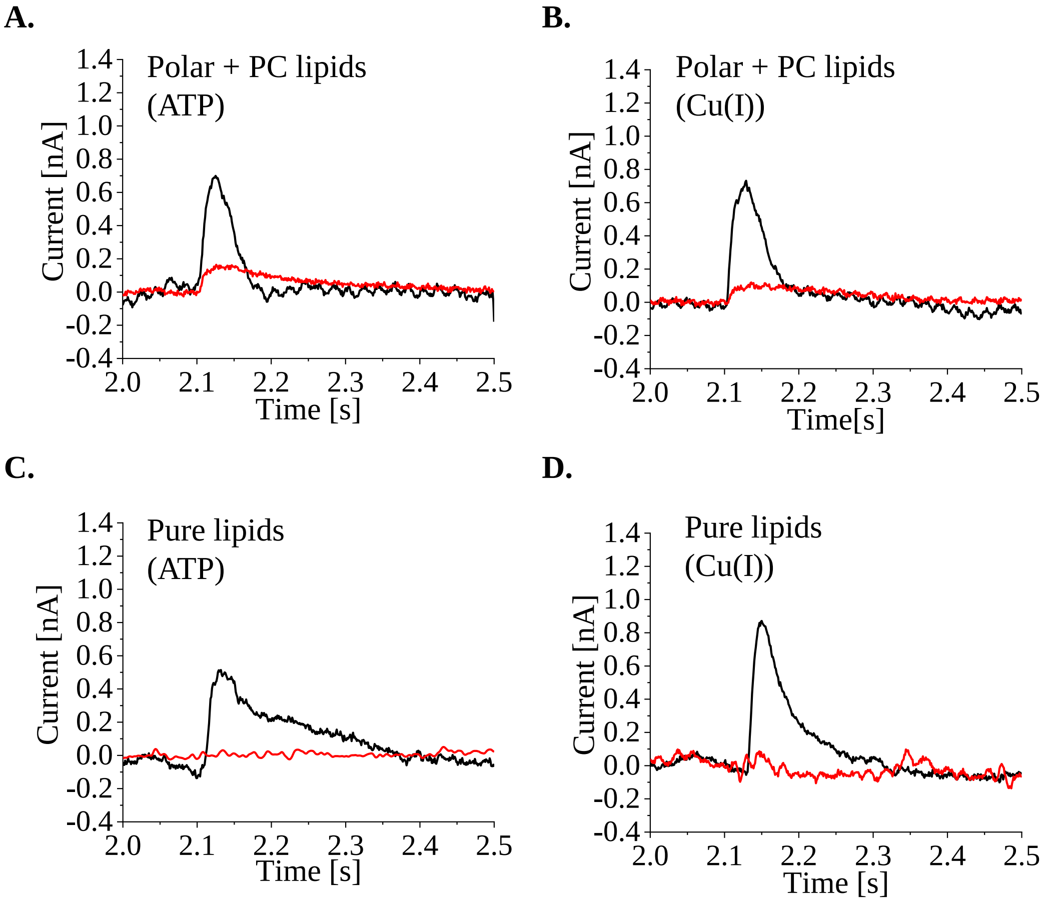

Figure S4: Optimization and comparison of experimental setups for LP and CcoI-LP in solid-supported membrane electrophysiology assays. Red and black lines indicate transient currents recorded for LP and CcoI-LP, respectively. In (**A**) and (**B**) vesicles were prepared using *E. coli* polar lipids extract enriched with phosphatidylcholine (Polar + PC). (**A**) Representative transient currents recorded using a concentration jump of 500 µM $CuCl_2$ in the A-solution with 250 µM ATP in the background and (**B**) a concentration jump of 250 µM ATP in the A-solution with 500 µM $CuCl_2$ in the background. In (**C**) and (**D**) vesicles were instead prepared using a 2:3.5:3.5:1 suspension of DSPC, POPE, SOPG and DGTS (pure lipids). (**C**) and (**D**) Representative transient currents obtained as described in (A), (B), respectively. The composition base of the A- and NA-solution here was 25 mM Tris pH 7.4, 300 mM KCl, 5 mM $MgCl_2$, 10 mM ascorbic acid, 20 mM cysteine and 500 µM $CuCl_2$ (or 250 µM ATP, as indicated in brackets).

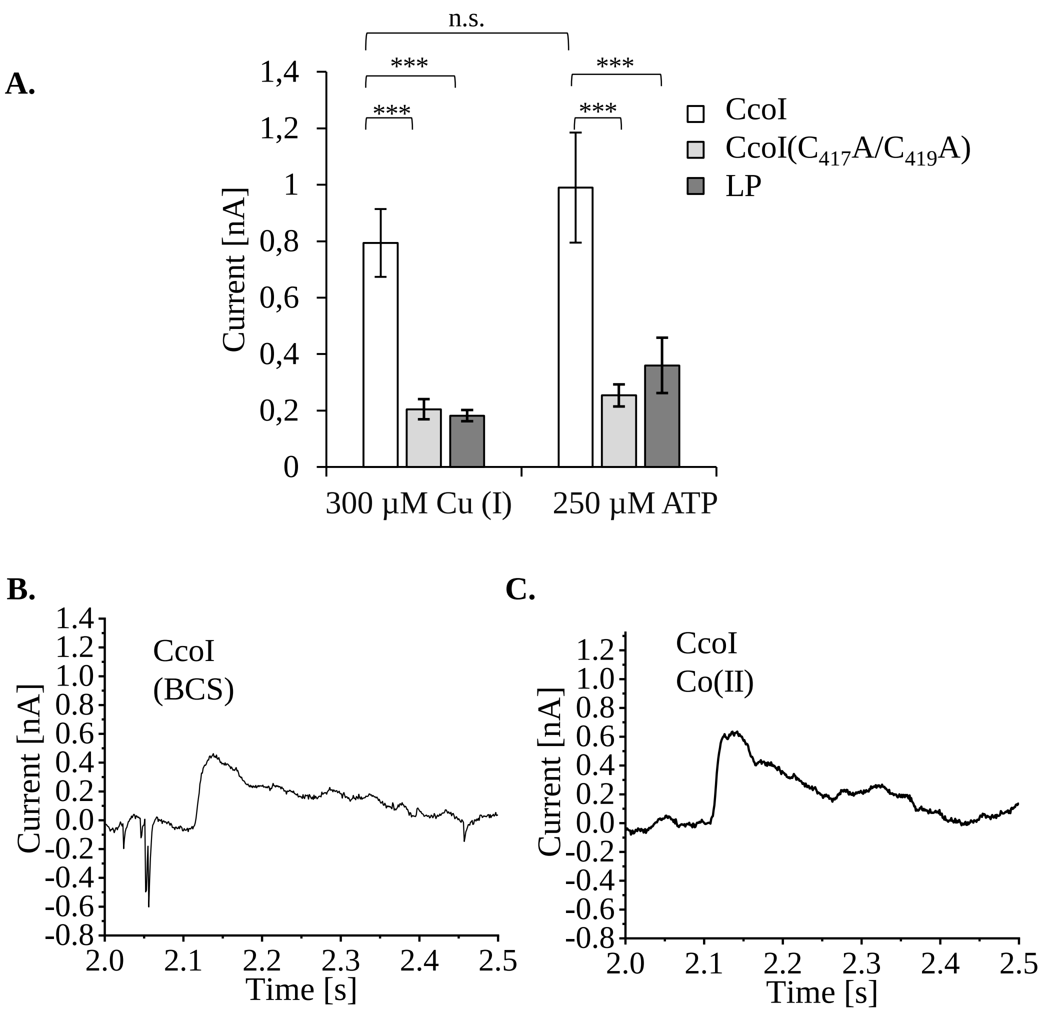

Figure S5: Electrogenic behavior of CcoI. (**A**) Average of transient current peak values obtained when wild-type CcoI or the CcoI(C_{417}A,C_{419}A) variant, reconstituted in lipid vesicles prepared in a 2:3.5:3.5:1 suspension of DSPC, POPE, SOPG and DGTS (pure lipids), were adsorbed onto sensor units, placed inside the SURFE^{2}R-N1 Faraday cage and subject to cycles of non-activating (NA-)/activating (A-)/NA-solution perfusions as described in the text. Currents were induced by adding either 300 µM Cu(I) or 250 µM ATP. The results obtained with liposomes (LP) account for the residual background artifacts, recorded in the absence of protein (negative control). Error bars reflect the SD and were calculated from 3 biological replicates for CcoI-LP (white bars), with 4 sensors per biological replicate each (n = 12); for CcoI(C_{417}A,C_{419}A) (light gray) the SD was calculated from 2 technical replicates with a total of 5 sensors (n = 5). For liposomes (LP, dark grey), SD was calculated from 2 technical replicates with 4 sensors each (n = 8). (*) refers to p-values ≤0.05; (**) to p-values ≤0.01, and (***) to p-values ≤0.001 (**B**) Representative transient currents acquired for CcoI-LP (pure lipids) upon a concentration jump of ATP (250 µM) in the A-solution with 5 mM BCS in the A- and NA-solutions. (**C**) Same as in (**B**) except that $CoCl_2$ replaced BCS in the A- and NA- solutions.

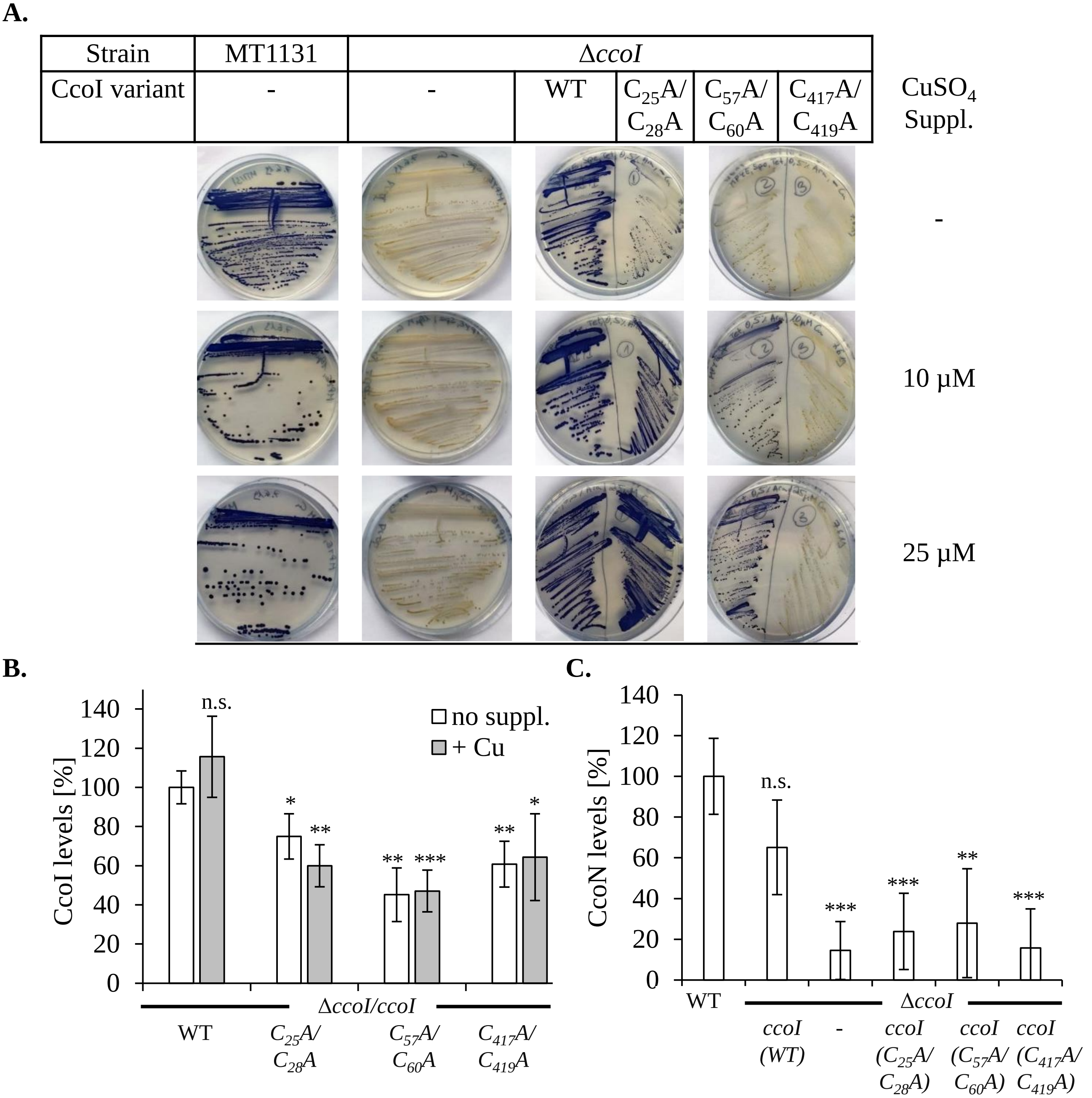

Figure S6: Distinct effects of the CcoI metal binding sites on *cbb*$_3$-Cox activity. (**A**) NADI staining of wild type *R. capsulatus* MT1131 or the Δ*ccoI* strain, expressing different plasmid-encoded *ccoI* variants. The strains were grown semi-anaerobically on MPYE media, supplemented with 10 µM, 25 µM $CuSO_4$ and without copper supplementation (-). 0.2% *L*-ara was added to the strains that carried a *ccoI* plasmid-borne copy to allow expression. Representative areas of the plates are shown in Fig. 4A. (**B**) Protein levels of CcoI and its variants produced in the Δ*ccoI* strain, grown either without Cu supplementation (white bars) or in the presence of 10 µM $CuSO_4$ (grey bars). A representative blot is shown in Fig. 4B. CcoI levels were determined by immune-detection using α-Myc antibodies and quantified via *Image J*. A representative gel is shown in Fig. 4C. The bars indicate the mean values of three biological replicates (total n = 4). The wild type CcoI levels of cells grown without Cu supplementation were set to 100% and used as reference for statistical analyses with the Satterthwaite corrected unpaired two-sided Students t-test. (*) refers to p-values ≤0.05; (**) to p-values ≤0.01, (***) to p-values ≤0.001 and (n.s.) to not significant. (**C**) The CcoN protein levels in wild type cells and in Δ*ccoI* cells expressing different CcoI variants (as in the representative blot in Fig. 4C) were detected by α-CcoN antibodies and quantified via *Image J*. The bars indicate the mean value of three biological replicates (total n = 5). The CcoN levels in wild type cells was set to 100% and used as reference for statistical analyses as in (**B**).

A.

- 10 μM 25 μM $CuSO_4$ Suppl.

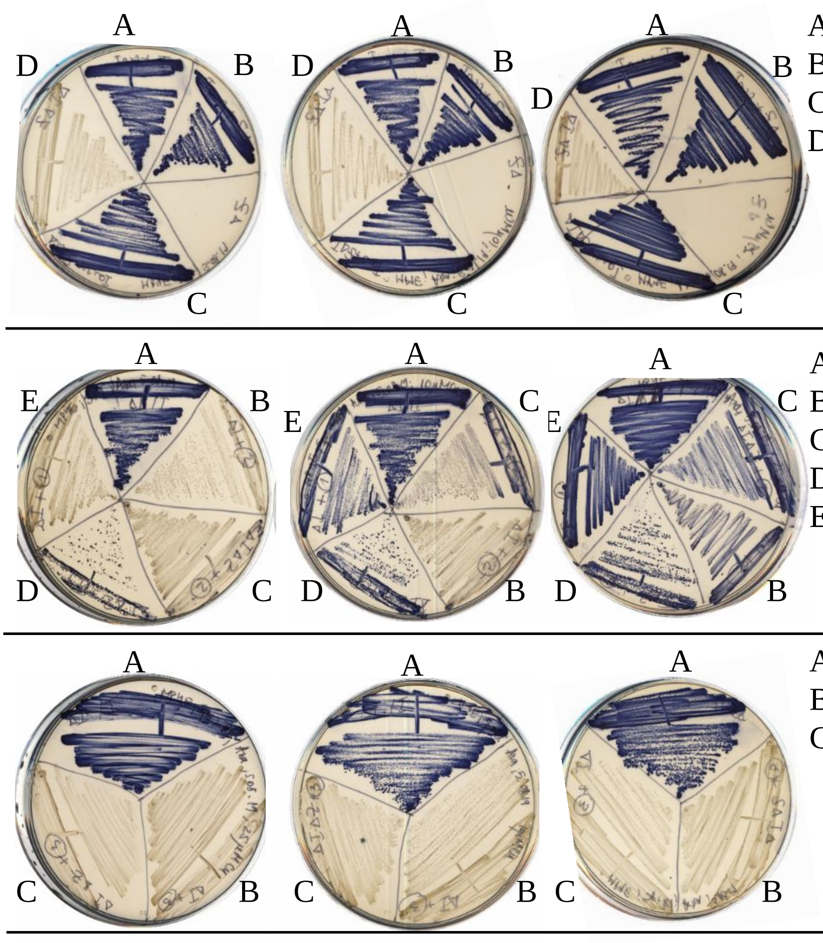

A- *ΔccoI/ccoI (WT)*
B- *ΔcopZ/ccoI (WT)*
C-*ΔccoIΔcopZ/CcoI*
D-*ΔccoIΔcopZ*

A- *ΔccoI/ccoI (WT)*
B- *ΔccoI/ccoI($C_{57}A/C_{60}A$)*
C- *ΔccoIΔcopZ/ccoI($C_{57}A/C_{60}A$)*
D- *ΔccoIΔcopZ/ccoI($C_{25}A/C_{28}A$)*
E- *ΔccoI/ccoI($C_{25}A/C_{28}A$)*

A- *ΔccoI/ccoI (WT)*
B- *ΔccoI/ccoI($C_{417}A/C_{419}A$)*
C-*ΔccoIΔcopZ/ccoI($C_{417}A/C_{419}A$)*

B.

Strain	*ΔccoIΔcopA*							
CcoI variant	WT		$C_{25}A/C_{28}A$		$C_{57}A/C_{60}A$		$C_{417}A/C_{419}A$	
Colony	1	2	1	2	1	2	1	2

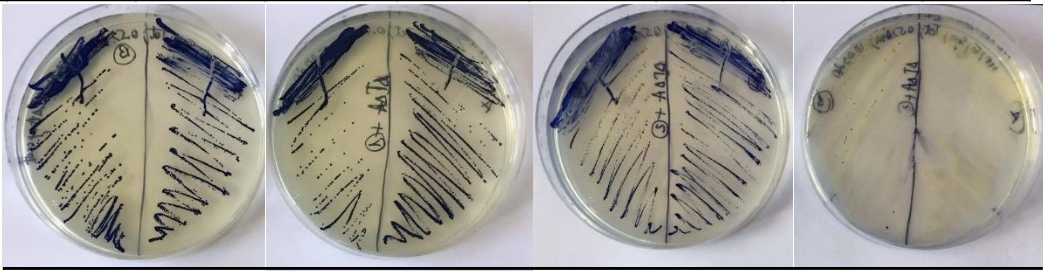

Figure S7: The cytoplasmic copper concentration influences metal acquisition by CcoI. (**A**) NADI staining of different *R. capsulatus* strains as indicated in the presence of 10 μM or 25 μM $CuSO_4$ and without copper supplementation. (**B**) NADI staining of the *R. capsulatus ΔccoIΔcopA* double-knock-out expressing different *ccoI* alleles, without Cu supplementation.

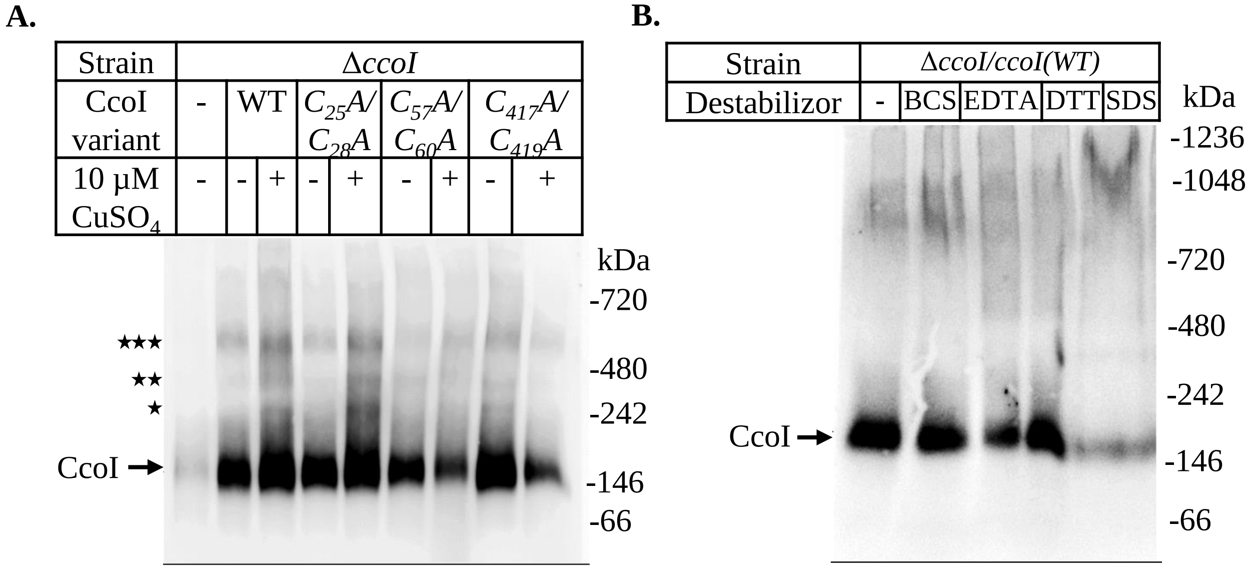

Figure S8: Blue native PAGE analyses of *R. capsulatus* CcoI. (**A**) Membranes of the *R. c.* Δ*ccoI* strain expressing the different *ccoI* variants and grown in the presence or absence of supplementary Cu were solubilized with 2% DDM, immunoblotted and treated with antibodies against the C-terminal Myc tag of CcoI. Δ*ccoI* was used as a negative control. The approx. 150 kDa band represents CcoI monomer. Three different unstable complexes are labeled with stars: (★) for the 230 kDa complex, (★★) for the approx. 400 kDa and (★★★) for the approx. 600 kDa complex. (**B**) One-hour solubilization of membranes from a *R. capsulatus* overproducing CcoI strain with 2% DDM was performed in the presence of 5 mM BCS, 5 mM EDTA, or 5 mM DTT at 4° C or 5% SDS at room temperature, for one hour. The complexes were loaded on BN-PAGE and treated as described above.
